# Transcriptome analysis of the bloodstream stage from the parasite *Trypanosoma vivax*

**DOI:** 10.1186/1471-2164-14-149

**Published:** 2013-03-05

**Authors:** Gonzalo Greif, Miguel Ponce de Leon, Guillermo Lamolle, Matías Rodriguez, Dolores Piñeyro, Lucinda M Tavares-Marques, Armando Reyna-Bello, Carlos Robello, Fernando Alvarez-Valin

**Affiliations:** 1 Unidad de Biología Molecular, Institut Pasteur de Montevideo, Montevideo, CP 11400, Uruguay; 2 Sección Biomatemática, Facultad de Ciencias, Universidad de la Republica Uruguay, Montevideo, Uruguay; 3 Departamento de Bioquímica, Facultad de Medicina, Universidad de la República Uruguay, Montevideo, Uruguay; 4 Centro de Estudios Biomédicos y Veterinarios, Universidad Nacional Experimental Simón Rodríguez-IDECYT, Caracas, Venezuela

## Abstract

**Background:**

*Trypanosoma vivax* is the earliest branching African trypanosome. This crucial phylogenetic position makes *T*. *vivax* a fascinating model to tackle fundamental questions concerning the origin and evolution of several features that characterize African trypanosomes, such as the Variant Surface Glycoproteins (VSGs) upon which antibody clearing and antigenic variation are based. Other features like gene content and trans-splicing patterns are worth analyzing in this species for comparative purposes.

**Results:**

We present a RNA-seq analysis of the bloodstream stage of *T*. *vivax* from data obtained using two complementary sequencing technologies (454 Titanium and Illumina).

Assembly of 454 reads yielded 13385 contigs corresponding to proteins coding genes (7800 of which were identified). These sequences, their annotation and other features are available through an online database presented herein. Among these sequences, about 1000 were found to be species specific and 50 exclusive of the *T*. *vivax* strain analyzed here. Expression patterns and levels were determined for VSGs and the remaining genes. Interestingly, VSG expression level, although being high, is considerably lower than in *Trypanosoma brucei*. Indeed, the comparison of surface protein composition between both African trypanosomes (as inferred from RNA-seq data), shows that they are substantially different, being VSG absolutely predominant in *T*. *brucei*, while in *T*. *vivax* it represents only about 55%. This raises the question concerning the protective role of VSGs in *T*. *vivax*, hence their ancestral role in immune evasion.

It was also found that around 600 genes have their unique (or main) trans-splice site very close (sometimes immediately before) the start codon. Gene Ontology analysis shows that this group is enriched in proteins related to the translation machinery (e.g. ribosomal proteins, elongation factors).

**Conclusions:**

This is the first RNA-seq data study in trypanosomes outside the model species *T*. *brucei*, hence it provides the possibility to conduct comparisons that allow drawing evolutionary and functional inferences. This analysis also provides several insights on the expression patterns and levels of protein coding sequences (such as VSG gene expression), trans-splicing, codon patterns and regulatory mechanisms. An online *T*. *vivax* RNA-seq database described herein could be a useful tool for parasitologists working with trypanosomes.

## Background

African trypanosomes, also known as Salivaria (acquiring this name because they complete the life cycle in the mouthparts or in salivary glands of the insect vector), are the causative agents of disease in humans, domestic and wild mammals. Some sub-species of *Trypanosoma brucei* species complex are responsible for producing the so called sleeping sickness in humans that affects thousands of persons each year in sub-Saharan African countries. *T*. *brucei*, along with other species of salivarian trypanosomes are the aetiological agents of a variety of livestock diseases not only in Africa, but also South America and Asia are affected by some species [1]. These cattle diseases, generally referred to as nagana, are accountable for important economic losses in the affected countries. Salivarian trypanosomes also infect wild animals (mostly ungulates), which may operate as natural reservoirs.

Apart from their African origin, other two distinguishing features of this group of trypanosomes are that they are mammalian parasites only and that their vectors are several species of the genus *Glossina* (tsetse flies). While in Africa Salivaria trypanosomes are transmitted both cyclically by tsetse flies and mechanically (i.e. without completing the cycle), in America only mechanical transmission by tabanids [2], other hematophagous fly species and even vampire bats has been observed [1,3]. It is not clear whether the ability to be transmitted mechanically by blood sucking insects other than tsetse flies is the ancestral transmission mode or a secondary adaptation to the particular environments that these parasites were exposed when they invaded African regions where *Glossina* was not present or new continents such as America or Asia. In this regard it is worth mentioning that early branching salivarians (like *T*. *vivax*) complete their cycle entirely in the proboscis of the fly (they cannot survive in the gut). This has been interpreted by Hoare (1972) [4] as a relict form, representing an intermediary stage in the evolutionary pathway from the ancestral mechanical transmission to full adaption to the salivary glands of tsetse fly.

However, the most remarkable adaptation of Salivaria trypanosomes is related to the fact that they remain exclusively extracellular in the mammalian host (in the bloodstream or in connective tissues), and hence permanently exposed to the immune system during infection. In all likelihood, such adverse condition is the reason (i.e. selective force) why in these parasites has evolved their most distinctive trait: a sophisticated strategy, called antigenic variation, to evade the host immune response. This strategy consists in periodically changing a dense protective coat composed by an extremely abundant (10^7^ copies) and immunogenic protein, the so-termed Variant Surface Glycoprotein (VSG). These parasites express only one VSG gene at a time, from a repertoire of silent copies that in the case of *T*. *b*. *brucei* contains more than 1500 different genes [5]. This mechanism allows transient immune evasion, since after changing the variable glycoprotein that was being expressed, an entirely new parasite population arises that is not recognized by the host's immune system which has developed an antibody response directed against the previous VSG. By repeating this cycle, the parasites are able to maintain the infection.

Reconstructions of evolutionary relationships using sequence data have shown that Salivaria trypanosomes are an indisputable monophyletic clade composed by three main groups [6]. These groups are basically in agreement with the traditional classification based on morphological and life cycle data proposed by Hoare (1972) [4]. The first group; which is fully coincident with Trypanozoon subgenus, contains the model species *T*. *brucei brucei*, the human pathogens *T*. *brucei gambiense* and *T*. *brucei rhodesiense* and two species of veterinary importance, *Trypanosoma evansi* and *Trypanosoma equiperdum*. A second group, the subgenus Nanomonas, includes two small sized nagana causing species, *Trypanosoma congolense* and *Trypanosoma simiae* (which are far more divergent to each other than is the divergence inside Trypanozoon). Finally, the Dutonella subgenus contains *Trypanosoma vivax*, another nagana causing species with economic importance, both in Africa and America. The use of suitable molecular markers on samples taken from the wild have recently disclosed that *T*. *vivax* also exhibits substantial intragroup diversity, comparable to that observed in *T*. *congolense*[7,8]. A relevant biological/evolutionary aspect of this last group, is that it occupies a crucial phylogenetic position because besides being the earliest branching Salivaria, its divergence predates that of the remaining ones by a big amount. This key evolutionary position, sometimes incorrectly referred to as being “the most primitive”, makes *T*. *vivax* a fascinating model to study fundamental questions concerning the origin and evolution of several features that characterize African trypanosomes. Indeed, the availability of data from *T*. *vivax* brings the possibility of making evolutionary inferences concerning the ancestral or derived state of relevant biological features by means of comparisons with *T*. *brucei* (or/and other salivarians) and consequently provides the opportunity of analyzing these traits in different stages of their evolution (as it has been mentioned before for the mode of transmission).

A recent evolutionary genomic analysis has been conducted in *T*. *vivax* and other representative species of African trypanosomes, comparing their repertoires of silent VSG genes, how they are organized and diverge aiming to understand the evolution of these proteins and how they gave rise to novel functions [9]. It was found that species differ in the organization of their silent VSG archive, something that may result in different mechanisms for generating antigenic diversity. Besides, these authors suggest that while in *T*. *brucei* and *T*. *congolense* there is a high rate of recombination between silent VSG copies, this phenomenon is much less pronounced in *T*. *vivax*. This analysis, however, barely addresses the topic of the expression of this fundamental group of proteins. In fact no previous genome wide studies on gene expression have been published on *T*. *vivax*. To tackle this and other important questions, we have conducted RNA-seq analyses of the bloodstream stage in *T*. *vivax* using different and complementary ultra-high throughput sequencing technologies. Deep sequencing in trypanosome species other than *T*. *brucei* may contribute to understand several topics concerning the biology of trypanosomatids (notably regulation of gene expression) by giving the possibility of conducting comparative analyses and providing an evolutionary perspective. Surprisingly, this technology has been scarcely used in trypanosomatids, being restricted to the model species *T*. *b*. *brucei* and more recently RNAseq has been used in *Leishmania tarentolae* to explore the role of the nucleotide *J* (β-D-glucosyl-hydroxymethyluracil) in transcription regulation [10].

## Methods

### Parasites

#### Experimental infection and parasite purification

*T*. *vivax* from the bovine Venezuelan isolate (LIEM-176) were used in this work.

Purification of trypanosomes was done as follows: immunosupressed six-months-old cross-bred sheep were inoculated intravenously with cryopreserved blood containing *T*. *vivax*. When parasitemia reached values of 2 x 10^7^ trypanosomes/ml, blood was extracted and mixed with an equal amount of Percoll (Sigma) containing 8.55% sucrose, 2.0% glucose, pH 7.4 and then centrifuged at 17000 g, 20 minutes at 4°C. Parasites were recovered from top and middle layer of Percoll gradient, resuspended in PBS (sodium phosphate 40 mM, pH 7.5, NaCl 150 mM) containing 1% glucose (PBSG) and subsequently centrifuged at 6000 g for 15 minutes at 4°C. The pellet containing parasites was washed twice with PBSG to remove residual Percoll. Partial purified parasites were resuspended in PBSG and applied to a DEAD-cellulose anion exchange column. Purified parasites were eluted free from red cells, examined by microscopy and counted in a Neubauer chamber. Further details can be found in [11]. *T*. *vivax* Y486 was grown on mice as described by Chamond et al. [12]. Briefly, 7 to 10-weeks-old male C57BL/6 mice were used. RNA and DNA samples for downstream analysis were obtained from 10^1^–10^5^ bloodstream forms obtained at the peak of parasitemia (day 8–10 post infection). Parasites were maintained by weekly passages in mice and new stabilates were appropriately and regularly frozen. All animal work was conducted in accordance with relevant national and international guidelines. Mice were housed in the animal care facilities from Institut Pasteur of Montevideo (Uruguay). Animal housing conditions and protocols used in the present work were previously approved by the CEUA (Ethical Committee for Laboratory Animal Use) under the number 013–11 according to the Ethics Chart of animal experimentation which includes appropriate procedures to minimize pain and animal suffering. Infections in sheep were conducted under veterinary supervision with daily control of temperature and hematocrit which never descended below 30%.

### RNA purification and quality control

Total RNA was isolated from 10^9^ parasites using Illustra RNAspin Mini Kit (GE Healthcare, USA) according to manufacturer’s protocol. Obtained RNA was quantified in a Nanodrop (Thermo Scientific, USA) and its integrity was checked by Bioanalyzer (Agilent, USA).

### Library construction and sequencing

Double-stranded cDNA was generated from 25 μg of total RNA using a SuperScript II Double-Stranded cDNA Synthesis Kit (Invitrogen) according to the manufacturer’s instructions, except for oligonucleotide used for first strand synthesis and 5-methyl-dCTP (Jena Biosciences) instead of dCTP. The primer used was 5^′^ CTGGAG(T)_16_VN 3^′^, the 5^′^ end of the primer contain the restriction site for the enzyme *GsuI*. After the synthesis of the second strand, the cDNA was precipitated with 1/10 volumes of Sodium Acetate (3 M, pH =5.2), 2 μL of glycogen (15 μg/mL) and 3 volumes of absolute ethanol and resuspended in 70 μL of RnaseFree water. 65 μL of cDNA was digested with *GsuI* (Fermentas) for 4 hours at 30°C to cleave the poliA tails. The digested cDNA was used to prepare the 454 and Illumina libraries.

### 454 library preparation and sequencing

Library was prepared using the GS Titanium DNA library preparation kit (Roche) according to the manufacturer’s protocol starting with 2.5 μg of cDNA. The emPCR was done with GS Titanium SV empPCR kit (Roche) according to manufacturer’s instructions. We used GS Titanium Sequencing Kit XLR70 (Roche) to sequence 1/2 GS Titanium PicoTiterPlate kit 70 × 75 in 454 Genome Sequencer FLX System (Roche). Illumina sequencing was carried out in a GAIIx on the same cDNA library which was re-fragmented and universal Illumina adaptors were added. Raw data were deposited in the NCBI database under submission number SRA056332.

### Bioinformatics and data analysis

#### Data quality analysis

The details of sequence data obtained by 454 and Illumina sequencing are presented in Additional file 1: Table S1. For the first technology 187491 reads, with an average length of 295 nt. were obtained. This corresponds to 54 Mb of sequence data. For the second technology, 37 million of reads (36 nt), corresponding to 1332 megabases were obtained. Several quality tests were carried out. In the first place, the percentage of contaminating reads present in the sample (i.e. corresponding to the host) was determined. For this purpose the reads were mapped into the sheep genome using Blastn. By doing this it was possible to establish that only 433 reads (i.e. 0.20%) were of host origin. A similar figure was obtained for Illumina reads (in this case mapped using Bowtie [13]). The same procedure was followed for other possible contaminating sources (such as human) and only traces were detected (e.g. 12 reads from 454 technology corresponding to human). This indicates that the quality of the starting material was high.

In the second place the number of artificially repeated reads (i.e. those corresponding to the cases when the same cDNA segment is sequenced more than once) were identified. This distortion (common in 454 sequencing) is introduced during the emulsion PCR step because a single cDNA molecule, but multiple beads are located in the same micro reactor. For genomic sequences these are customary identified as “same-start reads” provided that it is unlikely that by chance alone multiple DNA segments obtained by random fragmentation of a genome start at exactly the same position. However, it is obvious that for RNA derived DNA (cDNA), sharing the “same-start” is not uncommon. For this reason the candidates of artificially duplicated reads were identified as those ones that start and end at exactly the same nucleotide. About 15000 reads (9%) fall in this category (Additional file 1: Table S1), such proportion of repetitions is low when compared with other studies, where this kind of reads can be as abundant as 25%. These repeated reads were collapsed for further analysis.

In the next step, reads corresponding to ribosomal RNA were identified, totaling 2267 (1.21%) in the case of 454 FLX. The percentage of rRNA reads was significantly higher for Illumina (more than 2 million, which corresponds to slightly more than 6%). Such a small number is unusual considering that this type of RNA normally represents more than 70% of the RNA population, thus indicating that the filtering strategy of using an oligo-dT containing primer turned out to be very effective in order to get rid of ribosomal RNA. In addition, this methodology does not restrict the isolated RNA to mature mRNA either, as it can be inferred from the fact that other types of RNA molecules are quite abundant in the sample. In effect, transcripts derived from the kinetoplast genome are relatively abundant (Additional file 1: Table S1). For the case of maxicircle, it was possible to identify them using simple homology search, given that these genomes are relatively conserved among trypanosomatids. But, such strategy was not suitable for minicircle derived RNA identification because of their lack significant conservation. Therefore the incidence of this latter group was not determined.

To evaluate the genome coverage of RNA-seq data produced in this work, 454 and Illumina reads were mapped to genomic sequences (retrieved form GenBank) in order to estimate the sequencing depths of the top, middle and botton 1000 expressed genes. This was done using RNA-SeQC program [14], detailed results are presented in Additional file 2: Figure S1.

#### Assembling and functional annotation

Assembling of 454 reads was conducted using two different computer programs Mira [15] and Newbler (Roche, Switzerland). The two resulting assemblies were compared to each other, in order to assess their qualities and determine which one was more appropriate for subsequent analyses. The quality of the assembly was assessed by comparing the assembled contigs with a reference set containing well defined mRNA sequences. To assess quality, two variables were measured, the proportion of the reference mRNA that is well reconstructed (P) and the number of contigs falling in each mRNA reconstruction fraction (N), so that the overall quality of the assembly is given by: $Q=\sum_{i}N_{i}P_{i}$. This comparison was done using a reference set consisting of protein coding sequences available in GenBank that are putatively expressed in the bloodstream stage. These were identified on the basis that their *T*. *brucei* orthologs are unambiguously expressed in this stage. In turn, the latter condition was determined by testing which *T*. *brucei* protein coding genes are observed in the bloodstream EST collection. It should be noted that this collection was built using traditional Sanger sequencing from poly A + RNA, which due to the low sensitivity of the method, contains mainly unequivocally transcribed genes. The results obtained allowed us to draw two useful conclusions. In the first place Mira outperforms Newbler, yet by a narrow margin; provided that the contigs built by Mira reconstruct better the mRNA (i.e. the Q statistics is higher). Secondly, more than 92% of the putatively expressed mRNAs are tagged (either by contigs containing several reads, or by individual reads), hence indicating the 454 derived sequence dataset is a good picture of the transcriptional state of the parasite (Additional file 3: Table S2).

Functional annotation of RNA derived contigs was carried out using a set of complementary tools: ESTscan [16], Blast2Go [17], InterProScan [18] and AnEnPi [19]. In the first place, to identify *T*. *vivax* genes encoding proteins with a known or unknown function, it was necessary to obtain high quality virtual translation of contigs. This translation is not always the straightforward exercise of mechanically applying the genetic code to possible ORFs. Instead, contigs often contain serious translation problems derived from sequencing errors that may change the reading frame (frameshifts). To handle this complication the ESTscan program was used. This application employs a Hidden Markov Model (that uses the distribution of codons) to restore the correct frame by introducing indels. The program needs to be calibrated (trained) in such a way that it recognizes possible alteration in the ORFs on the basis of their statistical properties [16]. For training the ESTscan HMM, *T*. *vivax* coding and intergenic sequences were retrieved from public databases. After this step, functional annotation of the translated contigs was done combining the results of Blastp against nr NCBI (all non-redundant GenBank CDS translations plus other well curated databases) and a domain analysis based on Interproscan. Results of both sources were integrated using Blast2Go, which allows assigning GO terms to the entries by using simple annotation rules. Because B2G is quite conservative to assign ontology terms, the analysis was complemented with a simple Blastp search against translated nr NCBI. Besides the AnEnPi pipeline [19] was used on KEGG in order to predict possible metabolic pathway that are active in the bloodstream stage of *Trypanosoma vivax*.

### Determination of transcription levels

To determine the transcription levels we decided to use Erange [20] software on Illumina data. After cleaning low quality reads, the remaining reads were mapped to the *T*. *vivax* genome (retrieved from GenBank) using Bowtie [13] and allowing up to 1000 multimatches and up to 1 mismatch. RNA-seq Erange pipeline was used with minor modifications. It is important to take into account that in genomes like this one, which contain several related paralogous genes, the use of computer applications that consider the unique regions of the genes to re-normalize the assignment of multimatching reads (like Erange), is essential. This approach permits also determining which ones of the paralogous genes from a multigene family are really expressed at a given time. For 454 data (where the problem of multimatching reads is mitigated or simply eliminated, because of reads' lengths) transcription levels were computed using in house Bash and Perl scripts to parse Blast or Bowtie outputs. rpkm estimates are presented on Additional file 4: Table S3.

We note that in these analyses it was not possible to use biological replicates. Because of the limited amounts of RNA isolated in each individual infection, it was necessary to pool all samples together. Although this is not optimal because variability is not assessed, for a couple of reasons such limitation is not critical for this study. First, the main focus of our study is not compare different moments of the parasite life cycle aiming to determine which genes are up or down regulated. Moreover, since our starting material is a pool of different biological independent samples, large variance that might especially affect low expression genes (and yield a distorted picture) is largely alleviated. Transcript levels for *T*. *brucei* genes were also estimated as described above using published RNA-seq data [21] retrieved from the SRA archive.

#### Identification of splice-acceptor sites

cDNA sequence tags (36 bp) that contained terminal Spliced Leader sequence (SL) were extracted from the Illumina output. The SL sequence was found in a 0.5% (171200) of the reads and in the majority (94.8%) of them in the sense direction, as expected because of constraints imposed by the cDNA size-fractionation and sequencing protocols. The Spliced Leader segment was trimmed from these sequence tags with a homemade python script. Sequences greater than 19 bases were used in downstream pipeline. Genomic matches were identified by mapping these reads with Bowtie against the *T*. *vivax* genome sequence. No mismatches were allowed.

We used output of Bowtie (sam file), genomic information as given by the gff files and blockbuster software [22] to cluster the mapped reads in order to detect trans-splicing sites in the chromosomes and other genomic sequences from *T*. *vivax*. Cluster information was parsed with gff information with homemade Perl script and a final table with gene information and trans-splicing sites associated were obtained. A similar pipeline was used to analyze trans-splicing patterns in *T*. *brucei* and *Trypanosoma cruzi*.

In the case of *T*. *cruzi*, the RNAseq data from three stages of the life cycle of the parasite (epimastigote, trypomastigote and amastigote) were obtained in our laboratory (further analysis on this data will be published elsewhere). Due to the sequencing strategy used in *T*. *cruzi* (stranded) the number of Spliced Leader containing reads was modest; this restricted the type comparisons that could be conducted in this species to only the determination the splicing motifs.

## Results and discussion

### Assembling 454 reads and functional annotation of resulting contigs

Because 454 FLX sequencer yields long reads, it is possible conduct “de novo” assembling to obtain good quality contigs. This was done with two different computer programs, Mira and Newbler (Roche) using optimized parameters for RNA-seq assembling.

The results obtained allowed us to conclude that Mira outperforms Newbler, since the contigs obtained represent better reconstructions of full length mRNA (i.e. the Q statistics is higher). Besides, more than 92% of the putatively expressed mRNAs are tagged (either by contigs containing several reads, or by individual reads), hence indicating that the 454 derived sequence dataset is a good picture of the transcriptional state of the parasite (Additional file 3: Table S2).

As mentioned before high quality virtual translations of contigs were obtained using ESTscan. A total of 13385 translatable sequences were identified by ESTscan among which 6583 contained more than one read. Functional annotation, carried out using Blast2GO, enabled us to identify 3834 contigs for which it was possible to assign one or more Gene Ontology terms. However, the number of contigs whose virtual translation have homologs in other species (blast e-value <1e^-10^) was 2 times as much (7796), and hence it was possible to make a relatively reliable primary functional assignment for these contigs as well. In addition, we could determine a tentative enzymatic function using KEGG search for a substantial number of virtual translations totalizing 327 EC numbers assigned to 1281 contigs. Additional results on the functional annotation are available in the web page (see next section).

Finally, it is worth mentioning that more than 1000 contigs that are transcribed at different levels and unequivocally correspond to protein coding genes, do not have homologs in other species, including other trypanosomatids such as *Leishmania sp*, *T*. *cruzi* and the African trypanosomes for which genome sequence is available (*T*.*b*. *brucei*, *T*.*b*. *gambiense* and *T*. *congolense*). This means that in all likelihood they are species specific. Among these *T*. *vivax* specific contigs, around 50 genes have not even been reported in the *T*. *vivax* genome available in GenBank, indicating that very probably they are specific of the strain LIEM-176. 564 species specific contigs for which it was possible to build a full cDNA were chosen for additional analysis. A preliminary characterization of these genes was carried out using a battery of informatics tools such as those that identify signals for sub-cellular localization and domain analysis. These results are presented in Additional file 5: Table S4. Database web interface.

A relational database (MySQL) was built to store and browse the data and results produced in this work. In fact the database contains raw as well as processed and annotated data as described in the previous section. A Pyhton web application was developed using the Django programming framework. This application provides user-friendly data querying, browsing and visualization through a web interface (http://bioinformatica.fcien.edu.uy/Tvivax/). In this web interface it is possible to search for, and retrieve reads, contigs as well as virtual translations. Besides, the database can be searched using different criteria such as length, depth of the contigs (i.e. expression level), GO terms, Enzyme Commission numbers, Blast e-values, keywords, etc. or a combination of these criteria. Moreover any sequence can be blasted against the dataset. The annotated entries are linked to the reference databases used for their annotation, namely Amigo Gene Ontology [23], KEGG repository at EBI and NCBI. In addition the database offers the possibility to highlight *on*-*the*-*fly* the enzymes in the pathway image files downloaded from the KEGG FTP site. Expression of Variant Surface Glycoproteins in T. vivax, and the protein composition of the cellular surface.

Because of the strategic evolutionary position of *T*. *vivax*, as the earliest branching African trypanosome, it is important to analyze in this species the expression patterns of Variant Surface Glycoproteins, as well as the organization of this gene family to help shedding light on diverse questions concerning the origin and evolution of antigenic variation.

To this aim we first tried to identify the VSGs that were present in our RNA sample by using a simple strategy, which consisted in searching putative candidates among the most abundant mRNAs (namely those contigs built with the highest number of 454 reads), provided the high expression levels that these proteins exhibit. By doing this, only one candidate VSG was found. Surprisingly, the mRNA identified was highly similar (DNA sequence identity of 90.4%, see Additional file 6: Figure S2) to the only VSG sequence already reported for *T*. *vivax* that was derived from a West Africa isolate called Ildat 2.1 [24]. Even if this finding confirms previous reports that suggest that the American *T*. *vivax* (or more correctly *T*.*vivax*- like given the great intra taxon diversity inside Dutonella) is closely related to West African strains [25] some remarks should be made. It should be taken into account that *T*. *vivax* was introduced in America around 1850, in the French Guiana, by infected Zebus imported from Africa [26-29]. Since its introduction, *T*. *vivax* has been disseminated by horse flies (Tabanidae) [4] and stable flies (*Stomoxys* spp.) [30], and it was rapidly dispersed throughout South America. However, the degree of sequence similarity seems to be much higher than what we would have expected if account is taken to the fact that these genes normally diverges extremely fast. Indeed, the comparison of VSG genes among *T*. *brucei* strains reveals that even closely related subspecies (like *T*. *brucei brucei* and *T*. *brucei gambiense* and the so called Tororo isolates) have very divergent silent repertoires [31]. In addition, it should be noted that this VSG gene was not identified in the draft genome deposited in GenBank corresponding to the Y486 strain. We tested this absence by PCR using two sets of primers specifically designed to amplify this gene. Both primers sets failed to amplify, thus confirming that this VSG copy is really not present in the Y486 strain (Additional file 7: Figure S3). Conversely, the gene encoding the VSG protein expressed by Y486 (Ildat 1.2) is not detected in Liem-176 transcriptome. Considering that Y486 also belongs to the same group of West African *T*.*vivax*-like strains [7], these two results seem to be conflicting. Alternatively they indicate that the two processes of genetic differentiation of their silent archives, sequence divergence (involving single nucleotide changes) and genome plasticity (gene gain, loss and reshuffling) are not necessarily correlated, especially in this initial phase of taxa differentiation.

As far as the expression level of the main VSG is concerned, it is interesting to note that although its transcript abundance is very high (twice as much as the already highly expressed alpha and beta tubulins, see Additional file 8: Table S5), the number of Illumina reads mapping on this contig corresponding to the VSG gene, is not nearly as high as those reported for VSGs in *T*. *brucei*, where they represent between 5% and 11% of all sequenced reads [21,32,33]. This is an interesting aspect and raises several questions concerning the membrane protein composition and diversity (in terms of relative abundance of their constituent proteins) in this and other African trypanosomes.

**Figure 1:**
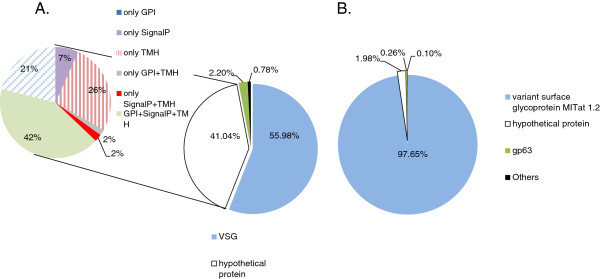
**Protein Membrane composition as inferred from expression levels. A**. *T*. *vivax*. **B**. *T*. *brucei*.

These questions can be answered by assessing the expression levels (as indicated by RNAseq data, see further details in next section) of genes encoding proteins predicted to have surface location, thus allowing us to compare the surface protein composition from both African parasites. As it emerges from Figure 1, it is evident that while in *T*. *brucei* the VSG is absolutely predominant (representing approximately 98%), in *T*. *vivax* it only represents about 55%. Other very common trypanosomatidae membrane proteins, like GP63, are almost absent in *T*. *brucei*, while they exhibit appreciable frequencies in *T*. *vivax*. These results thus indicate that the cellular surface of *T*. *vivax* is substantially different from that of *T*. *brucei* (and very likely from other African trypanosomes). In turn, these results concerning the much lower membrane concentration of VSG proteins are in keeping with previous ones from electron microscopy [34], which indicate that in *T*. *vixax* the VSG surface coat is noticeably less dense than in *T*. *brucei*. In addition, these results taken together raise the question of what would be the role of VSGs in *T*. *vivax* (and perhaps its ancestral role) in immune system evasion, provided that such relative lower concentrations cast some doubts on how efficiently it could act as a fully protective coat as it happens in *T*. *brucei*. Needless to say, proteomic analysis will provide more substantial data to help gaining additional insight on this fundamental point. In particular it would be important to analyze the efficiency of antibodies targeted against invariant membrane epitopes. Assessing transcription levels.

One of most useful features of RNA-seq analysis is that it allows direct and quite accurate estimations of transcript levels. Given that the number of reads matching with the transcripts of a given gene is expected to be proportional with the concentration of the mRNA molecule as well as with its length. Then the normalized (by length) numbers of 454 reads used for assembling of a given contig, or the number of Illumina reads mapping on the same contig (or on the corresponding genomic CDS) can be used as a measure of expression level.

**Figure 2:**
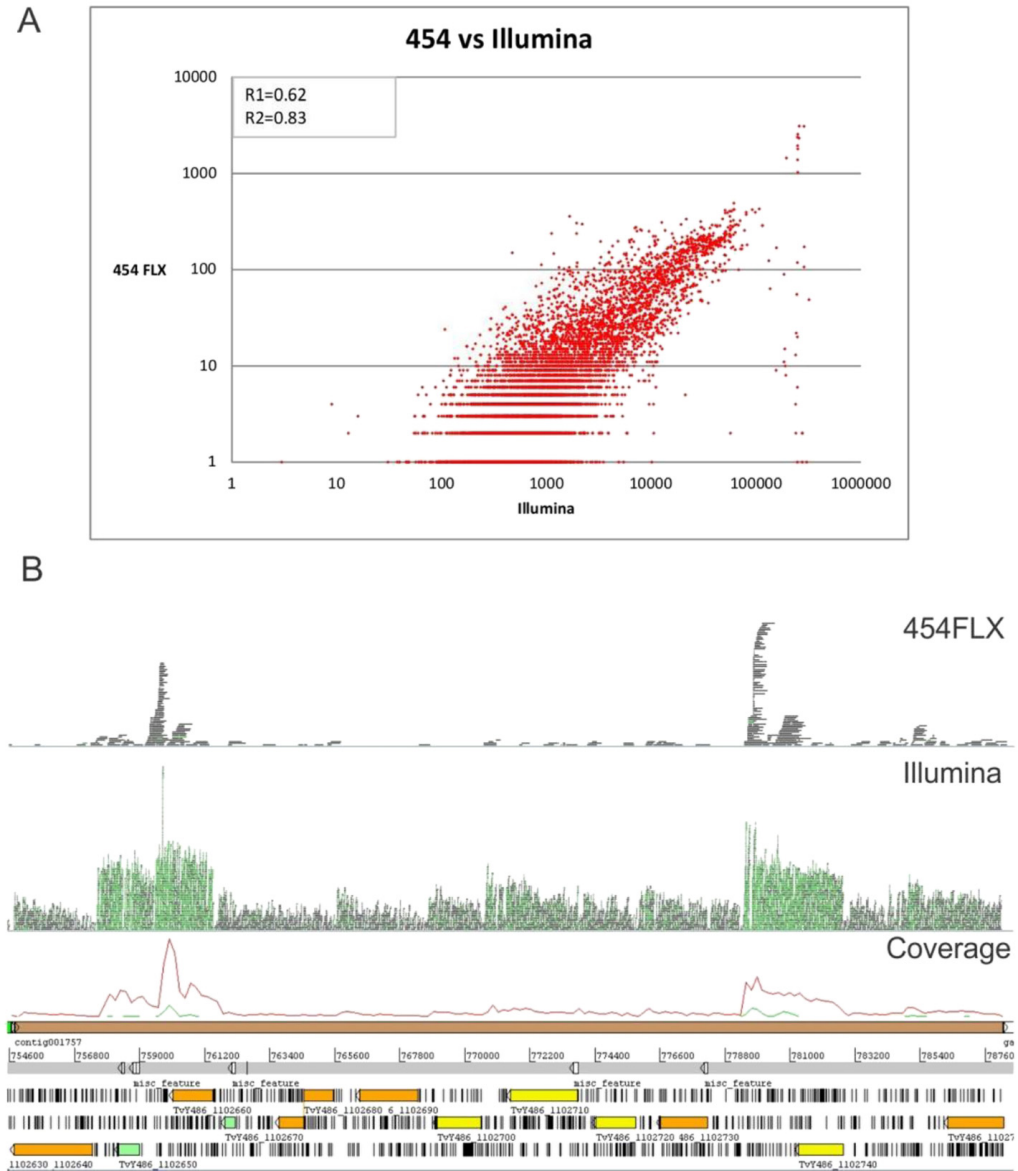
**Comparison of transcription levels estimation. A**. Scaterplot of the number of 454 FLX reads used in the assembly of a given contig versus the number of Illumina reads mapping on the same contig. R1 and R2 stand for the correlation coefficients before and after disregarding the points that exhibit an extreme discrepancy between the two technologies (those ones forming an almost vertical line on the rightmost part of the figure). **B**. This figure depicts a depth profile showing the reads that map on a given genomic region. The upper part corresponds to 454 FLX, Illumina reads appear in the middle and the last part corresponds to the graphical representation of the corresponding genomic region.

In the first place we compared how congruent are the two sequencing technologies used in this work for estimating transcript levels. Specifically, we compared the number of 454 FLX reads used in the assembly of a given contig versus the number of Illumina reads mapping on the same contig. As it can be observed in Figure 2, even though for some points (contigs) the estimation differs, the agreement is quite remarkable (r = 0.83). The genes (contigs) exhibiting estimations that are inconsistent between the two technologies were further analyzed to understand the reasons why these two technologies yield contradictory estimations. Indeed, for several genes very few 454 reads contributed to their assembly, while many of the same genes were tagged by considerable number of Illumina reads. Even if it is reasonable that many low expression genes that are tagged by Illumina reads will be not detected by 454 FLX sequencing technology, given the comparatively small number of reads the latter technology yields, in few cases the disagreement between the two technologies goes far beyond than what would be expected by random variability. In effect, since the ratio in the numbers of reads between the two technologies is 181 (see Additional file 1: Table S1), which is close to the regression coefficient in Figure 2A, it follows that several genes on which map many thousands of Illumina reads (>10 thousands) are not expected to be tagged by none or so few 454 reads. The visual inspection of these troublesome points shows that they correspond to DNA segments having extreme compositional biases. On the other hand the comparison between the two technologies was also conducted by mapping their reads on genomic regions to assess the variability in sequencing depths estimated by each method. Again it is possible to observe that there is a good agreement between both methods (Figure 2B).

Estimation of transcription levels for 11886 CDS annotated in GenBank was done using the Erange software that corrects multiple matching reads considering unique parts of genes for their assignation. The gene expression levels (read count and RPKMs) are available in Additional file 4: Table S3.

**Figure 3:**
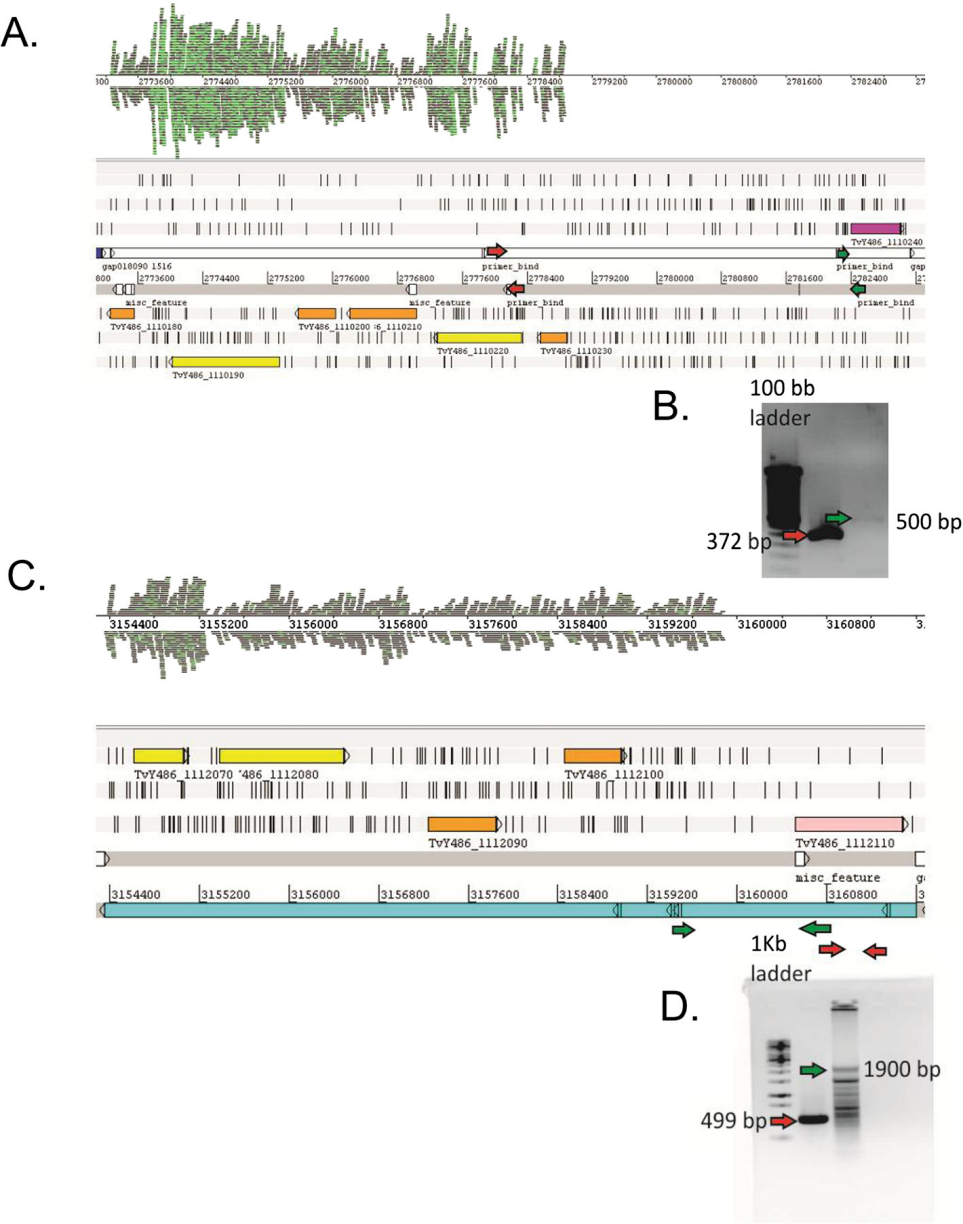
**The figure shows two representative genomic regions that appear to be not transcribed in bloodstream stage of *T. vivax *(A,C). B** and **D** PCR amplification of the genomic region represented in panels **A** and **C** respectively. The amplification confirms their presence in the genome of LIEM-176 strain. Arrows (red and green) represent the two primer sets used for each genomic region.

An unexpected observation is that several genes and genomic regions appear to be non-transcribed at all in the bloodstream stage of *T*. *vivax*, as it can be appreciated in Figure 3 panels A and C, which shows that some of these regions are devoid of reads. We note that this result could be attributed to the fact that the reference genome used to map reads and the RNA used in this work come from different *T*. *vivax* strains (Y486 and LIEM-176 respectively), thus the absence of reads in some regions could be simply the result that the regions in question are not present in Liem-176. To control this possibility, we decided to test if the genomic DNA segments without reads mapping on them are also present in the genome of the strain from where the RNA comes. Primers specific for these regions were designed (indicated in red and green in Figure 3B). The PCR results presented in Figure 3C indicate that the regions with no reads are definitively present in the LIEM-176 strain, and hence the absence of reads mapping on them is in all likelihood the result of their lack of transcription. These results have implications on the long standing questions concerning the mechanisms of gene expression regulation in trypanosomatids. Indeed, the current accepted view is that in trypanosomatids everything (or almost everything) is promiscuously transcribed, and they regulate their gene expression mainly post-transcriptionally, either by differential RNA maturation and degradation, or by controlling translation initiation or even post-translationally [35]. Hence, the results presented in Figure 3 showing that certain genes and genomic regions are not transcribed, strongly suggest that regulation of transcription initiation might also play an important role in gene regulation. Gene expression levels and codon biases in trypanosomes.

It is well established that in most organisms synonymous codons are not randomly used [36,37]. Biased codon usage may result from a diversity of factors, among which translational efficiency (translational selection) is one of the most important, being related to the fact that the preferred codons in highly expressed genes are recognized by the most abundant tRNAs. More than fifteen years ago, we have shown that in trypanosomatids there is enormous intragenomic variability in codon biases, and this was essentially the result of the interplay between mutational biases and translational selection. In this analysis it was also shown that, in the African trypanosome *T*. *brucei*, the putatively highly expressed genes exhibit essentially the same kind, but with lesser strength, of codon biases as in *T*. *cruzi* (towards G and C ending codons) [38]. One of the main drawbacks of these analyses, is that the data on expression levels were very fragmentary or simply assumptions (for instance we assumed that proteins like ribosomal proteins, elongation factors, and enzymes from glycolysis were highly expressed). Interestingly, some of these results were confirmed more recently, yet no analysis was carried out so far comparing codon preferences using robust data on gene expression [39]. The availability of NGS data gives the opportunity to re-address this topic from a more reliable perspective.

**Figure 4:**
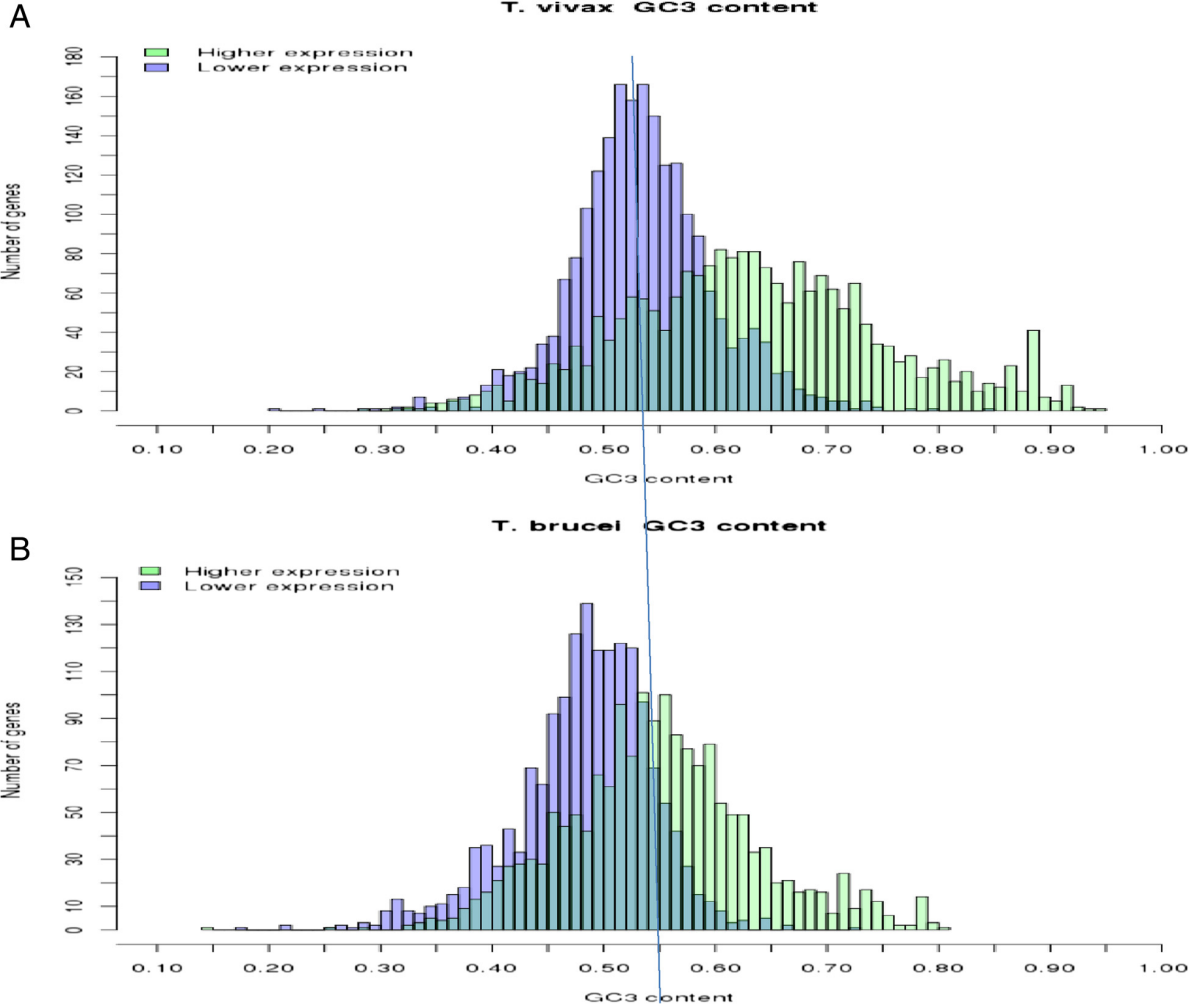
**Comparison of frequencies of G** **+** **C ending codons in the 20% most and least expressed genes in *T. vivax *(A), *T. brucei *(B).**

Figure 4A, shows the frequencies of G + C ending codons in the 20% most and least expressed genes in *T*. *vivax*. Even if it is possible to see that there is substantial variability inside each group, it is also clear that there is a very strong preference for G- and/or C-ending codons in the majority of genes that are more actively transcribed, and this preference also holds when each synonymous codon group is considered separately (Additional file 9: Figure S4). In agreement with previous results, it is possible to observe that also in *T*. *brucei* the highly expressed genes exhibit clear preference for G- and C-ending codons. However two differences should be pointed out. First, the overall distribution in GC_3_ values is shifted towards the left (namely lower values), and second the difference in GC_3_ preference between low and high expression genes is less pronounced than that observed in the other trypanosome. Based on these results it is possible to conclude that the process of weakening of codon biases observed nowadays in the high expression genes from *T*. *brucei* only affected the branch leading to the Trypanozoon subgenus, and not all Salivaria trypanosomes, provided that *T*. *vivax* did not undergo such a process.

An interesting observation is that in both African *Trypanosoma* species there is a group of genes that exhibit an atypical behavior in the sense that they are expressed at high or very high levels, yet they display weak or none GC_3_ biases. Furthermore, in both species the respective groups of unusually behaving genes include many species specific proteins and also proteins like many ribosomal proteins and translation factor 5a (well known to be highly expressed in most species). In addition, the two groups contain many genes that are coincident (i.e. orthologs) between *T*. *vivax* and *T*. *brucei* (Additional file 10: Table S6). It should be noted that the very existence of several orthologous that are highly expressed and lack codon biases in both species suggests that this unusual behavior cannot be attributed to natural variability in codon preferences that could eventually display high expression genes. Instead, this very likely reflects genuine functional requirements. We note that this peculiar observation had been pointed out before for the case of VSG genes in *T*. *brucei*, the highest expressed gene, yet the different genes encoding VSG proteins have very weak codon bias [38,40]. The puzzling aspect of this observation is why and how is it possible that these organisms do not optimize the codon preferences in genes that represent such a substantial proportion of the protein mass. Two different explanations (yet not mutually exclusive) can be put forward. One of these is that these genes belong to multigene families that have emerged, or became highly expressed, only recently (on an evolutionary scale). Hence selection did not have enough time to optimize their codon biases. This can be the case of leucine-rich repeat protein in *T*. *vivax*, procyclins in *T*. *brucei* (and also Mucin Associated Surface Proteins (MASP) in *T*. *cruzi*), that are very highly expressed yet have AT or weak GC biases (see Additional file 10: Table S6). The second explanation is that translational selection is not effective enough for these genes because they are seldom expressed, namely they behave most of the time as silent ORFs (like pseudogenes), during which time natural selection does not have any effect on them. This second explanation applies to VSG coding genes. Some additional analyses give support to these proposals. Indeed, when the analysis of the relationship between codon biases and gene expression levels is restricted to those coding sequences that have bona fide (and conserved) orthologs in other trypanosome species (what could be called the trypanosome gene core), most genes are “well-behaved”, that is the differences in GC codon biases between highly and lowly expressed genes become sharper in both species (Additional file 11: Figure S5).

Finally, we would like to mention that in spite of the fact that these explanations may account in part for this atypical behavior displayed by trypanosomes, it is also evident that they do not apply to the case of ribosomal and other conserved proteins that exhibit low o none GC_3_ biases and very high expression. Mapping trans-splice sites.

**Figure 5:**
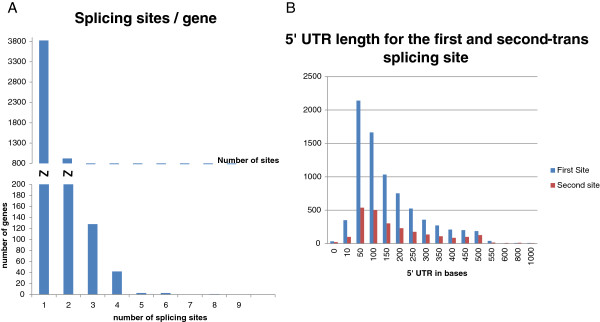
**Distribution of the number of trans**-**splicing sites and 5**^′^**UTR lengths. A**. Number of splicing sites per gene. **B**. Histogram of 5^′^UTR lengths to the first (FTSS) and second trans-splicing site (STSS). 5^′^UTR lengths average values 132 nts and 164 nts for FTSS and STSS respectively.

To identify trans-splice sites, we mapped 159395 miniexon containing Illumina reads onto the *T*. *vivax* draft genome that has been recently made available in Genbank. This allowed us to identify the trans-splice sites in 5959 genes. Among these genes, 3350 had only one bloodstream splice site and 2609 genes had two or more. The distribution of splicing sites per gene is presented in Figure 5A. The maximum number of sites per gene was 9 and the average 1.48. This figure is considerably lower than that described for *T*. *brucei* (mean 2.7-2.9 sites/gene [32]). Using the splice site location, the distribution of 5^′^ UTR lengths was also determined (Figure 5B). The mean sizes for the first and second splice sites were 132 and 164 nts, respectively. These are in the same range as it has been described for *T*. *brucei*[21,32,33].

**Figure 6:**
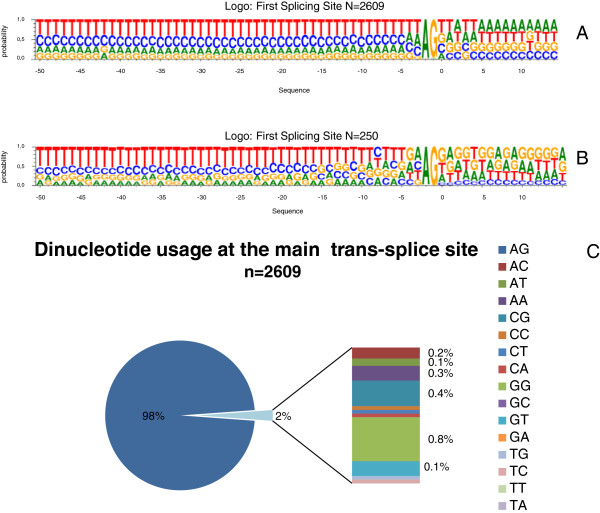
**Trans**-**splicing sites consensus sequence.** Panels **A** and **B**, logo representation of the major trans-splicing site (−50 nt and +15 nt relative to splicing site) from *T*. *vivax* (**A**), *T*. *cruzi* (**B**). **C**. Dinucleotide usage at the first tran-splicing site from *T*. *vivax*.

Next we analyzed the consensus sequences around the splice site. Figure 6A, shows the logo representation of the major site, which is virtually identical to that described for *T*. *brucei*, basically consisting in a long (>50 nt) poly-pyrimidine rich track. The consensus for the second and the remaining minor sites are also very similar yet the signal is not as strong as for the major site (Additional file 12: Figure S6) Furthermore, the canonical AG dinucleotide was found at 98% of the major splice sites (Figure 6C), whereas minor sites had an AG dinucleotide in progressively decreasing proportions, 94% for the second, 90% for the third and 80% for the fourth site. Therefore, the frequency of AG at secondary splice sites is considerable higher than that observed in *T*. *brucei* (that on average is around 80%, see reference [33]). As it has been also observed in *T*. *brucei*, the second most frequent dinucleotide at splice site is GG (Figure 6). A similar analysis was carried out also in *T*. *cruzi*; the logo illustration presented in Figure 6B shows that also in the American parasite the overall pattern is very similar to that of salivarians.

These results indicate that both the trans-splicing machinery, and the signals that this machinery recognizes, have been conserved not only in African trypanosomes, but also in *T*. *cruzi*, and therefore in all likelihood in all trypanosomes.

**Figure 7:**
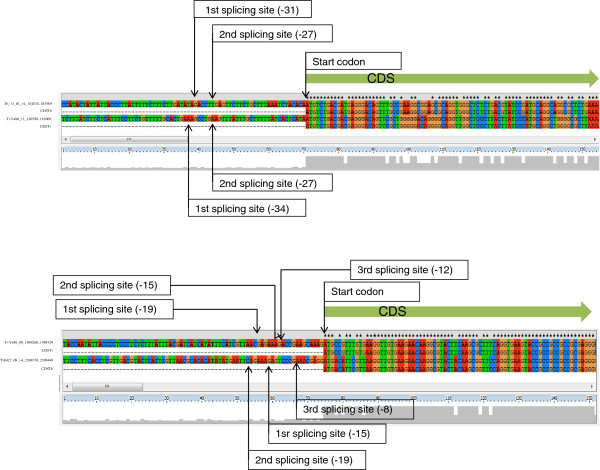
**Representative examples of 5**^**′**^**UTR distance conservation.** In the two examples (**A** and **B**) the distance to the different splice sites is fairly the same in both species, while sequence conservation in the 5'UTR is low.

Along the same line, we also compared orthologous genes between *T*. *brucei* and *T*. *vivax* to investigate whether the spatial pattern of trans-splicing sites, namely their number and distances to the initiation codon, was similar between these two African parasites. Interestingly enough, the pattern exhibited considerably agreement in spite of the fact that the DNA sequences in the 5' UTR located between the sites of splicing and the initiation codon were poorly conserved (Figure 7).

**Figure 8:**
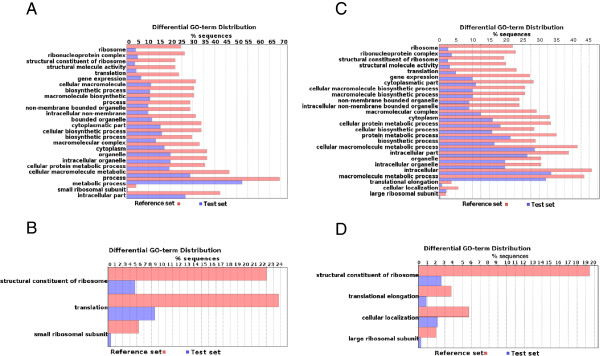
**Gene Ontology Enrichment Analysis.** These figures show the distribution of GO terms exhibiting statistical significant differences (Fisher Exact Test, filtering p-values for multiple testing using False Discovery Rate). The test set consisted in genes containing splicing site close to start codon (distance < = 10 nucleotides). Panels **A** and **B**: *T*. *vivax* In this case the testing set of short 5'UTR containing genes is composed by 196 sequences. The two panels correspond to different ontology levels (abstraction levels). Panels **C** and **D** show the equivalent GO analysis in *T*. *brucei* (n = 654).

As it has been already reported for *T*. *brucei*, a large number of *T*. *vivax* genes contain one or more (up to five) trans-splicing sites inside the coding region [21]. Moreover, we also found that a significant number of genes contain their main, or unique, splice site very close (sometimes immediately before and sometimes after) the start codon (AUG). We decided to investigate this peculiar aspect further by determining if this feature is characteristic of some groups of genes or functions. A Gene Ontology enrichment analysis was carried out to explore this aspect, namely if the genes exhibiting this feature encode proteins belonging to some particular categories. Interestingly enough, this group contains a much higher than expected frequency of ribosomal proteins, elongation factors and other proteins related with the translation machinery. Other type of proteins over-represented in this group are heat shock proteins and proteins that interact with RNA (Figures 8A and B).

Because the annotation of *T*. *vivax* genes available in GenBank is not precise in relation to the correct identification of start codons, and considering that this trouble can introduce serious biases in this analysis, the same ontology analyses were also conducted in *T*. *brucei*, whose annotation is expected to be much more depurated. As it can be observed in Figures 8C and D, the same pattern is also present in *T*. *brucei*, and hence allows us to conclude that it cannot be attributed to an artifact due to low quality annotation.

For these genes with splice site very close to the start codon, we identified the orthologs between *T*. *vivax* and *T*. *brucei*, and in many cases the splice sites were located upstream of the annotated start codon in one of the species but downstream in the other. We suspected that in all likelihood this was caused by the above mentioned trouble of misidentified start codons. Therefore their sequences were aligned to determine, on the basis of DNA and amino acid conservation, the most probable start codon. For these comparisons sequences from *T*. *congolense* (whenever available) were also included. The rationale for this approach for detecting more accurately AUG start codons is simple, and it is based on the fact that inside the coding part of the genes there are higher functional constraints and hence higher conservation. The approach allowed us to detect that many AUG codons were incorrectly annotated as the starting ones not only in *T*. *vivax* but also (and unexpectedly) in *T*. *brucei*. After correcting the annotation using conservation information, it was possible to determine that almost all downstream splice sites have in fact an upstream location (see Additional file 13: Figure S7 for representative examples). In addition when the orthologs between these two species are compared in relation to this feature, it is possible to observe that there is a very good agreement, namely the number of splice sites and their distances to the initiation codon is roughly the same (Additional file 14: Table S7). Noteworthy, while these distances remain, there is very little sequence conservation between the two species in the 5' UTR, which strongly suggests that what it is important is indeed the distance and not the sequence. Regretfully this analysis could not be extended to *T*. *cruzi* due to the limited number of reads that spanned the trans splicing junctions and retained a big enough sequence (>15 nt) after the Spliced leader was removed.

Although the biological significance of these observations is not fully clear, some hypotheses could be advanced on why this particular group of genes contain so short 5' UTR. It has been proposed that highly expressed genes tend to be more compact, shortening their 5' and 3' UTRs and introns to reduce energetic cost of protein synthesis [41]. At a first glance this explanation appears to fit the results presented herein provided that ribosomal proteins are normally highly expressed. However, the average expression level (as indicated by their transcript abundance) of short 5'UTR containing genes is not significantly different to the average expression level of the genome (*T*-test, p < 0.05).

Alternatively this feature could be related to genes that are constitutively expressed. This hypothesis becomes clearer if two aspects are taken into consideration; first translation initiation plays a key role in trypanosomatid expression regulation, and second it has been demonstrated in *Leishmania* that the sole presence of a Spliced Leader ensures the recruitment of the 40S ribosome complex to the mRNA 5^′^ (through the eIF4F initiation complex binding to the 5^′^ m7G-mRNA cap and/or to the SL itself) [42]. Therefore the lack of a segment between the Spliced Leader and the start codon (to which negative regulators could eventually bind), would imply that once the ribosomal initiation complex is assembled, there is almost no chance of blocking translation initiation. In this regard it is worth mentioning that it has been recently proposed that trypanosomes may contain posttranscriptional cis-regulatory elements located in the 5^′^ UTRs, which would be part of a mechanism to sense environmental changes (temperature) in a way reminiscent to bacterial RNA thermometers [35]. At any rate, the results presented here give initial hints that would require additional experiments (e.g. constructs containing specific modifications in the 5^′^ UTR) to test this or alternative hypotheses.

## Conclusions

In this work we conducted a RNA-seq analysis in *T*. *vivax*, a species of great importance for comparative purposes owing to its evolutionary location as the earliest branching African trypanosome. To this aim we sequenced the bloodstream stage of its life cycle using two complementary sequencing technologies. The first of these technologies allowed us to obtain a high quality assembly without the restriction of a reference set. The annotation of the contigs thus obtained (using a battery of bioinformatic tools) permitted the identification of about 6500 protein coding genes and other non-coding RNAs. Noteworthy, more than 1000 genes were found to be species specific and about 50 exclusive of *T*.*vivax* LIEM-176. This information and the partial reconstruction of metabolic pathways, is publicly available through a searchable online database.

The use of Illumina technology in combination with the above mentioned assembly and genomic information was used to analyze several aspects in this species which in turn allowed us to draw relevant conclusions by means of comparative analysis with *T*. *brucei*.

One first aspect to be emphasized concerns the Variable Surface Glycoproteins, that exhibit levels of expression considerably lower than those observed in *T*. *brucei*; an observation that is consistent with previous indications obtained from microscopy. This denotes not only that the proteins composition of cellular surfaces is notably different between the two species; but also implies that in all likelihood the way VSG proteins accomplish their shielding role did not remain exactly the same since their emergence. In this regards it is worth reminding that in *T*. *brucei*, the VSG coat is a dense physical barrier around the parasite, which does largely modulate the ability of immunoglobulins to recognize other surface (invariant) proteins. This point, which is of chief importance to understand the primordial function of VSGs, requires further investigation on diverse aspects such as assessing the level of exposure to the immune system of *T*. *vivax* invariant surface proteins or determining their efficiency in antibody clearing and the VSG switching rate.

As long as the expression patterns is concerned, we would like to stress that we present in this work evidence that some regions of *T*. *vivax* genome (that contain coding genes) have no transcriptional activity. In fact, a detailed study shows that vast genomic regions encompassing about one third of the repertoire of variant genes and other regions containing other protein coding sequences are transcriptionally inactive (Lamolle et al., in preparation). This strongly suggests, in contrast to the generally accepted view, that in trypanosomatids the regulation of transcription initiation might also play an important role in gene expression regulation. This works perhaps by switching off and on entire genome segments, something that might be accomplished by different mechanisms like condensation or loosening of chromatin in specific regions.

Finally, we would like to address the topic of trans-splicing patterns exhibited by *T*. *vivax*. A first conclusion that can be drawn in relation to this topic, is that the signals recognized by the trans-splicing enzymatic machinery (and thus the machinery itself) are substantially conserved not only in African trypanosomes but also in most distant species like *T*. *cruzi*. Another significant aspect is that the distance distribution of trans-splice sites, but not the sequence, is conserved for an important proportion of genes. The last important point regarding trans-splicing, is that a group of genes related to translation and interaction with RNAs, contain very short 5'UTR (i.e. the splice site is located just before the start codon). This observation cannot be attributed to any technical (bias in library preparation, sequencing) or bioinformatic (determination of AUG codon) artifact provided that the same pattern is found in both *T*. *brucei* and *T*.*vivax*. Although here we suggest some possible explanations and hypotheses that are in line with the regulatory role already proposed for the 5^′^UTRs in trypanosomatid RNAs, additional data from other trypanosomatid species will allow to determine the phylogenetic extent of this feature; and experiments (such as the use of manipulated DNA segments) would help shed light on its possible functional role.

## Competing interests

Authors declare that they have no competing interests.

## Authors’ contributions

GG and GL performed library preparation and sequencing. MPL and GL conducted the assembling of 454 contigs. MPL worked in the de annotation contigs. MPL and MR developed the online database and several Perl and Python scripts. MR took care of codon usage analysis and Perl and Python scripting. GG, MPL, MR, FAV were in charge of bioinformatic analysis (SL location, determination of expression level, splice leader analysis). GG, DP, LTM and ARB were in charge of experimental infection, parasite purification and RNA isolation. ARB was the veterinary that took care of sheep health condition. CR and FAV conceived the work. GG and FAV wrote the manuscript. All authors read and approved the final version of the manuscript.

## Supplementary Material

Additional file 1: Table S1 Details of sequence data obtained from 454 FLX and Illumina. Click here for file

Additional file 2: Figure S1 Coverage Metrics for Top-Middle-Lowest 1000 Expressed Transcripts. Click here for file

Additional file 3: Table S2 Quality of Assembly. Excel table containing Q values obtained by mira and Newbler assemblers. Click here for file

Additional file 4: Table S3 Expression levels. Excel table containing expression levels (rpkm values) obtained with erange. Comparison between 454 and Illumina quantification. Click here for file

Additional file 5: Table S4 Species specific proteins. Sheet 1. List and features of the contigs. Sheet 2. Summary table. Click here for file

Additional file 6: Figure S2 Sequence alignment of VSG from American and African isolates. Click here for file

Additional file 7: Figure S3 PCR of VSG. Genomic amplification with VSG specific primers in American and African isolates. Click here for file

Additional file 8: Table S5 rpkm and percentage of total sequence reads corresponding to VSG and tubulin genes in *T*. *vivax* and *T*. *brucei*. Click here for file

Additional file 9: Figure S4 GC_3_ content discriminated by amino acid. Click here for file

Additional file 10: Table S6 Group of genes having high expression levels (rpkm > average, 3 SD) and low GC3 frequency. MASP and ribosomal genes in *T*. *cruzi*. Click here for file

Additional file 11: Figure S5 Comparison of frequencies of G + C ending codons in the most and least expressed genes in *T*. *vivax* and *T*. *brucei*. The comparison was done between conserved and non conserved orthologous genes (up and low panels). Click here for file

Additional file 12: Figure S6 Sequence logo representation of 2nd to 4th trans-splicing sites. Click here for file

Additional file 13: Figure S7 Examples of Trans-splicing sites in *T*. *brucei* and *T*. *vivax* and annotation correction. Click here for file

Additional file 14: Table S7 *T*. *vivax* and *T*. *brucei* orthologs genes and trans splicing sites. Click here for file
