# Supplementary material for: Transcriptome analysis of the bloodstream stage from the parasite Trypanosoma vivax
Source: BMC Genomics. 2013 Mar 5;14:149. doi: 10.1186/1471-2164-14-149 (PMC4007602; doi:10.1186/1471-2164-14-149)
Supplement: Additional file 1: Table S1 — Details of sequence data obtained from 454 FLX and Illumina. [file 1471-2164-14-149-S1.doc]

**Table S1.**

Details of sequence data obtained from 454 FLX and Illumina.

|  | **454 FLX** | **Illumina** |
| --- | --- | --- |
| **Number of Reads** | 187128 | 37406418 |
| **Average Read Length** | 289 | 36 |
| **Number of Reads after eliminating low quality** | ----- | 34128677 |
| **Artificially Repeated Reads** | 15000 | ----- |
| **Host contamination** | 445 (0.20%) | 166000 (0.48%) |
| **Reads corresponding to Ribosomal RNA** | 8385 (4.48%) | 2035269 (5.95%) |
| **Reads correspoding Maxicircles** | 8587 (4.58%) | 778146 (2.28%) |
| **Reads with SL** | 3022 (1.61%) | 171239 (0.5%) |
