# Supplementary material for: Transcriptome analysis of the bloodstream stage from the parasite Trypanosoma vivax
Source: BMC Genomics. 2013 Mar 5;14:149. doi: 10.1186/1471-2164-14-149 (PMC4007602; doi:10.1186/1471-2164-14-149)

**Figure S1.**

**Illumina Coverage Metrics for Top 1000 Expressed Transcripts**

The metrics in this table are calculated across the transcripts that were determined to have the highest expression levels.

| **Sample** | **Note** | **Mean Per Base Cov.** | **Mean CV** | **No. Covered 5'** | **5'200Base Norm** | **No. Covered 3'** | **3' 200Base Norm** | **Num. Gaps** | **Cumul. Gap Length** | **Gap %** |
| --- | --- | --- | --- | --- | --- | --- | --- | --- | --- | --- |
| [Tv](file:///C:\EQC\illu\Tv\highexpr\index.html) | RNAseq | 216.25 | 0.52 | 982 | 0.75 | 986 | 787 | 46707 | 4.9 |  |

**
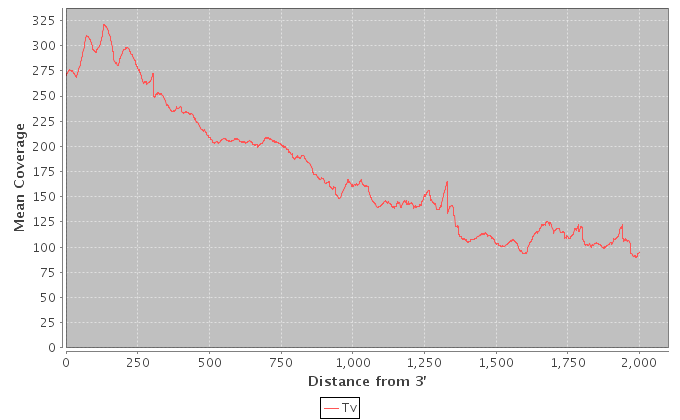
**

**
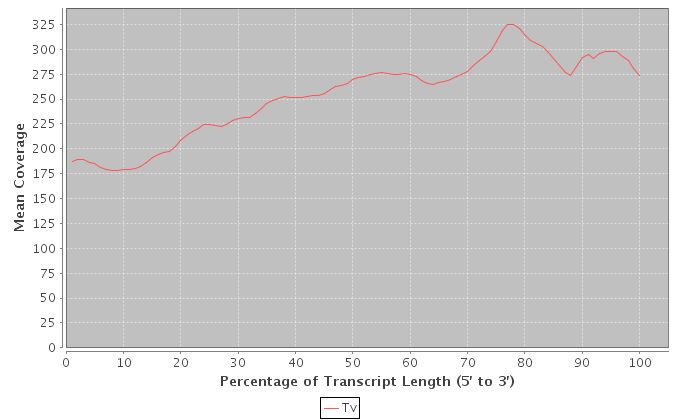
**

**Illumina Coverage Metrics for Middle 1000 Expressed Transcripts**

The metrics in this table are calculated across the transcripts that were determined to have the highest expression levels.

| **Sample** | **Note** | **Mean Per Base Cov.** | **Mean CV** | **No. Covered 5'** | **5'200Base Norm** | **No. Covered 3'** | **3' 200Base Norm** | **Num. Gaps** | **Cumul. Gap Length** | **Gap %** |
| --- | --- | --- | --- | --- | --- | --- | --- | --- | --- | --- |
| Tv |  | 17.28 | 0.42 | 997 | 0.76 | 999 | 387 | 11599 | 0.8 |  |


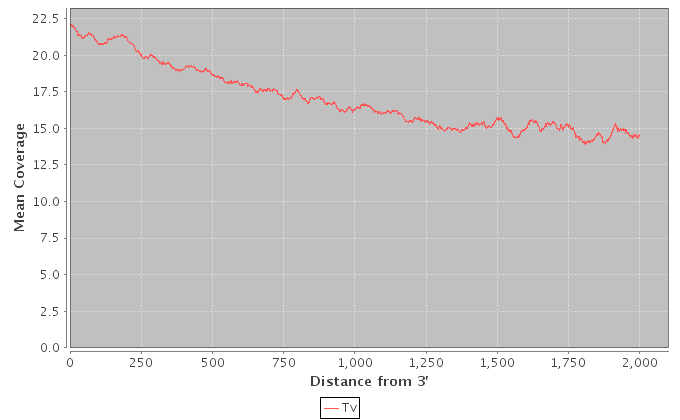


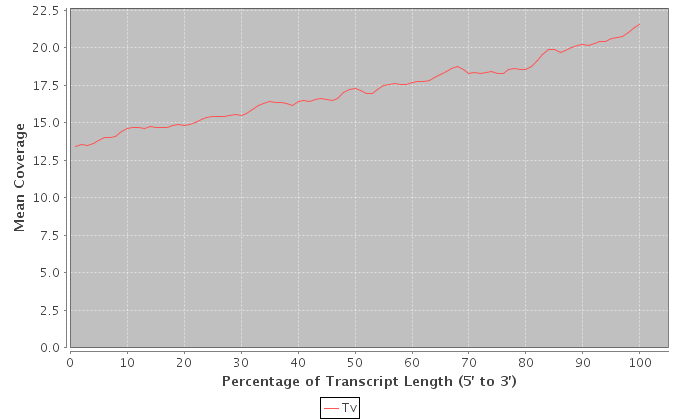


**Illumina coverage Metrics for Bottom 1000 Expressed Transcripts**

The metrics in this table are calculated across the transcripts that were determined to have the highest expression levels.

| **Sample** | **Note** | **Mean Per Base Cov.** | **Mean CV** | **No. Covered 5'** | **5'200Base Norm** | **No. Covered 3'** | **3' 200Base Norm** | **Num. Gaps** | **Cumul. Gap Length** | **Gap %** |
| --- | --- | --- | --- | --- | --- | --- | --- | --- | --- | --- |
| [Tv](file:///C:\Documentos\Datos\Gonzalo\IPMON\Proyectos\Vivax\Illumina\RNAseqQC\illumina\Tv\lowexpr\index.html) | RNAseq | 6.11 | 0.95 | 855 | 1.04 | 843 | 3636 | 188898 | 15.4 |  |

**
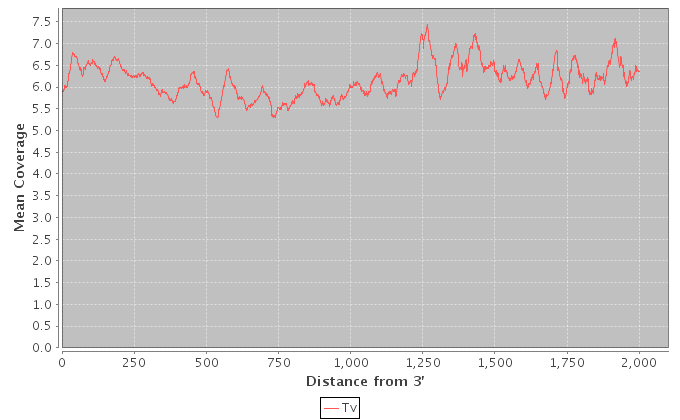

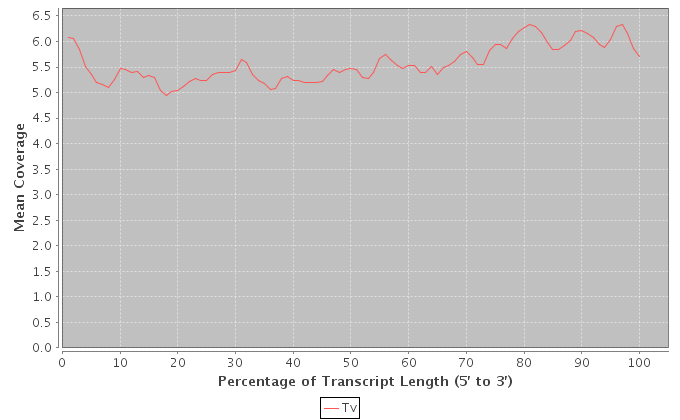
**

**454 FLX Coverage Metrics for Top 1000 Expressed Transcripts**

The metrics in this table are calculated across the transcripts that were determined to have the highest expression levels.

| **Sample** | **Note** | **Mean Per Base Cov.** | **Mean CV** | **No. Covered 5'** | **5'200Base Norm** | **No. Covered 3'** | **3' 200Base Norm** | **Num. Gaps** | **Cumul. Gap Length** | **Gap %** |
| --- | --- | --- | --- | --- | --- | --- | --- | --- | --- | --- |
| [Tv](file:///C:\EQC\Tv\highexpr\index.html) | RNAseq | 6.83 | 0.62 | 806 | 0.72 | 856 | 661 | 120692 | 13.2 |  |


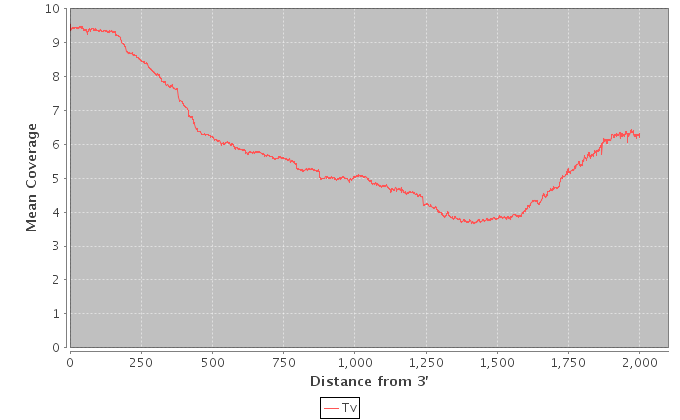

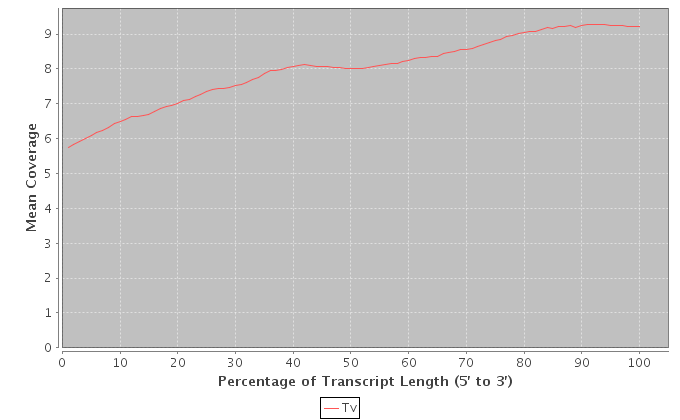


**454 FLX Coverage Metrics for Middle 1000 Expressed Transcripts**

The metrics in this table are calculated across the transcripts that were determined to have the highest expression levels.

| **Sample** | **Note** | **Mean Per Base Cov.** | **Mean CV** | **No. Covered 5'** | **5'200Base Norm** | **No. Covered 3'** | **3' 200Base Norm** | **Num. Gaps** | **Cumul. Gap Length** | **Gap %** |
| --- | --- | --- | --- | --- | --- | --- | --- | --- | --- | --- |
| [Tv](file:///C:\EQC\Tv\medexpr\index.html) | RNAseq | 0.90 | 1.05 | 418 | 0.84 | 528 | 2016 | 691519 | 45.0 |  |


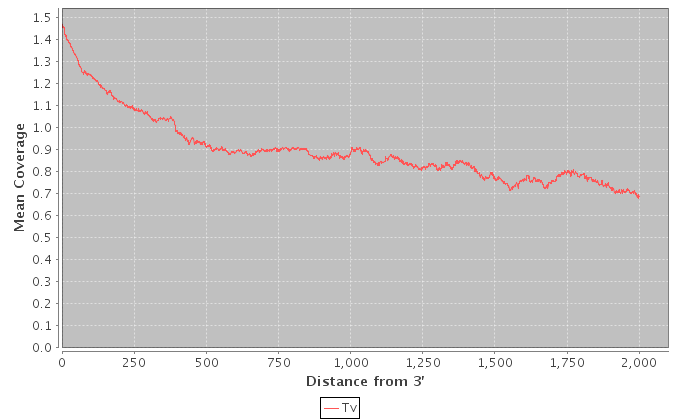


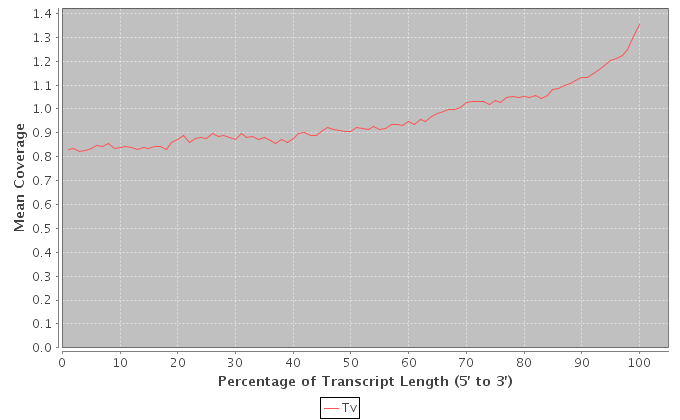


**454 FLX Coverage Metrics for Bottom 1000 Expressed Transcripts**

The metrics in this table are calculated across the transcripts that were determined to have the highest expression levels.

| **Sample** | **Note** | **Mean Per Base Cov.** | **Mean CV** | **No. Covered 5'** | **5'200Base Norm** | **No. Covered 3'** | **3' 200Base Norm** | **Num. Gaps** | **Cumul. Gap Length** | **Gap %** |
| --- | --- | --- | --- | --- | --- | --- | --- | --- | --- | --- |
| [Tv](file:///C:\Documentos\Datos\Gonzalo\IPMON\Proyectos\Vivax\Illumina\RNAseqQC\bwa\454bwa\Tv\lowexpr\index.html) | RNAseq | 0.28 | 2.15 | 200 | 1.47 | 311 | 1977 | 1649691 | 76.6 |  |


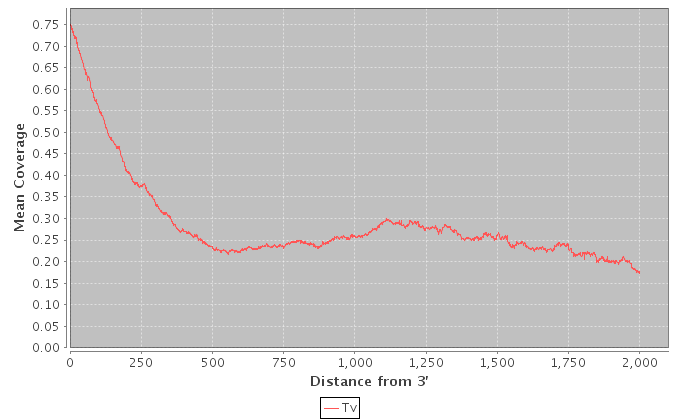

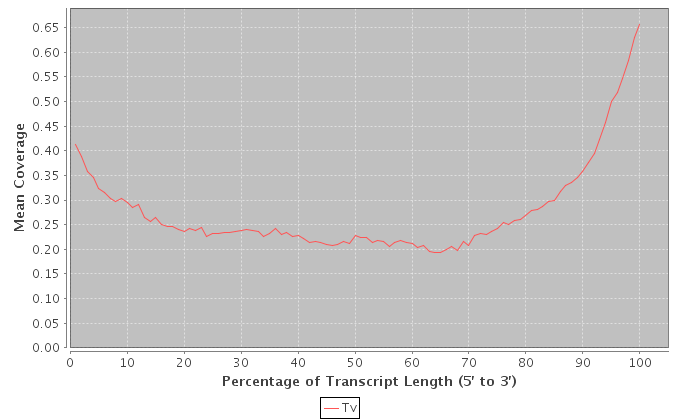

Supplement: Additional file 2: Figure S1 — Coverage Metrics for Top-Middle-Lowest 1000 Expressed Transcripts. [file 1471-2164-14-149-S2.docx]
