# Supplementary material for: Transcriptome analysis of the bloodstream stage from the parasite Trypanosoma vivax
Source: BMC Genomics. 2013 Mar 5;14:149. doi: 10.1186/1471-2164-14-149 (PMC4007602; doi:10.1186/1471-2164-14-149)
Supplement: Additional file 6: Figure S2 — Sequence alignment of VSG from American and African isolates. [file 1471-2164-14-149-S6.pdf]

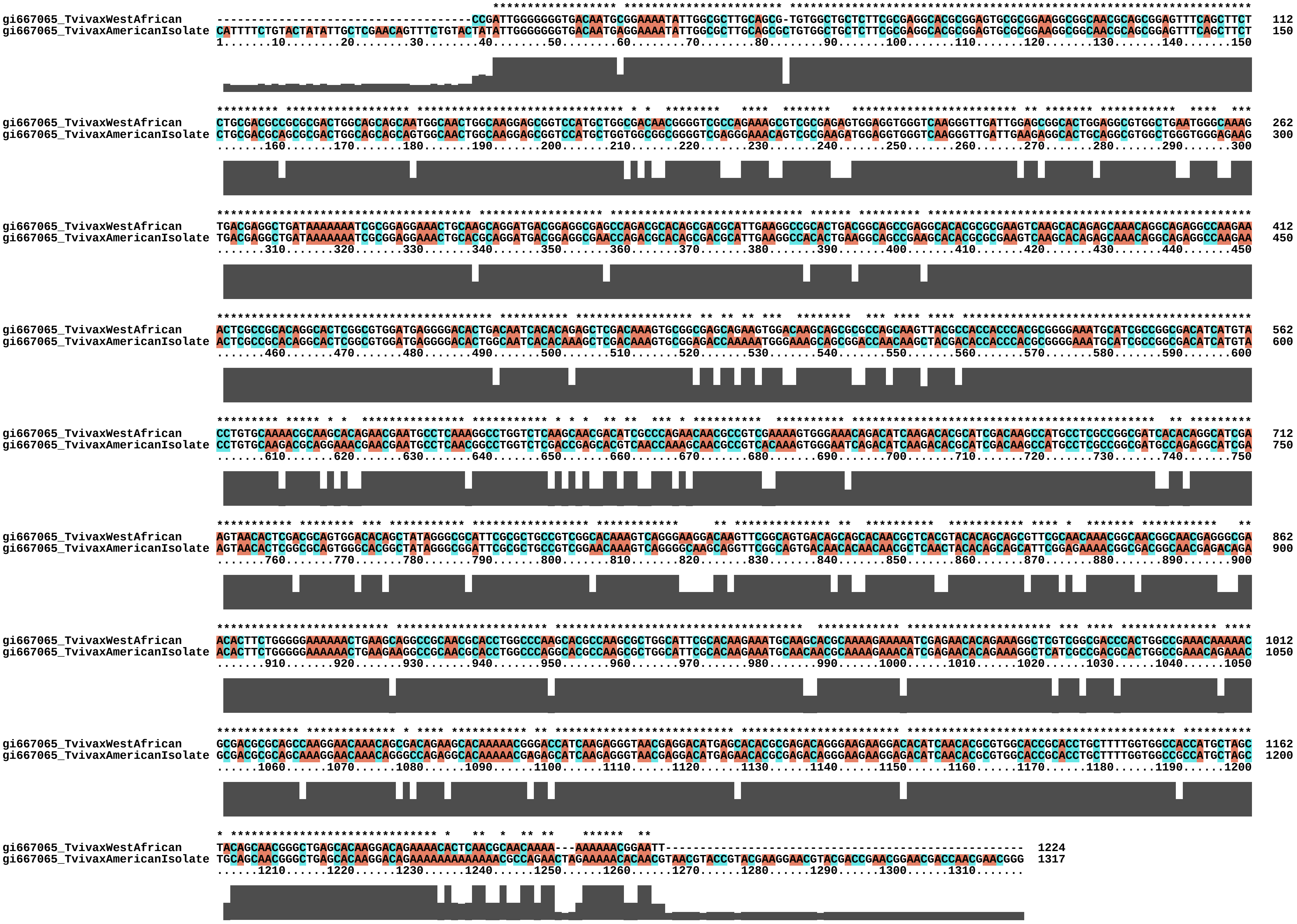
gi667065_TvivaxWestAfrican
gi667065_TvivaxAmericanIsolate
112
150
262
300
412
450
562
600
712
750
862
900
1012
1050
1162
1200
1224
1317
