# Supplementary material for: Transcriptome analysis of the bloodstream stage from the parasite Trypanosoma vivax
Source: BMC Genomics. 2013 Mar 5;14:149. doi: 10.1186/1471-2164-14-149 (PMC4007602; doi:10.1186/1471-2164-14-149)
Supplement: Additional file 7: Figure S3 — PCR of VSG. Genomic amplification with VSG specific primers in American and African isolates. [file 1471-2164-14-149-S7.ppt]

## Slide 1
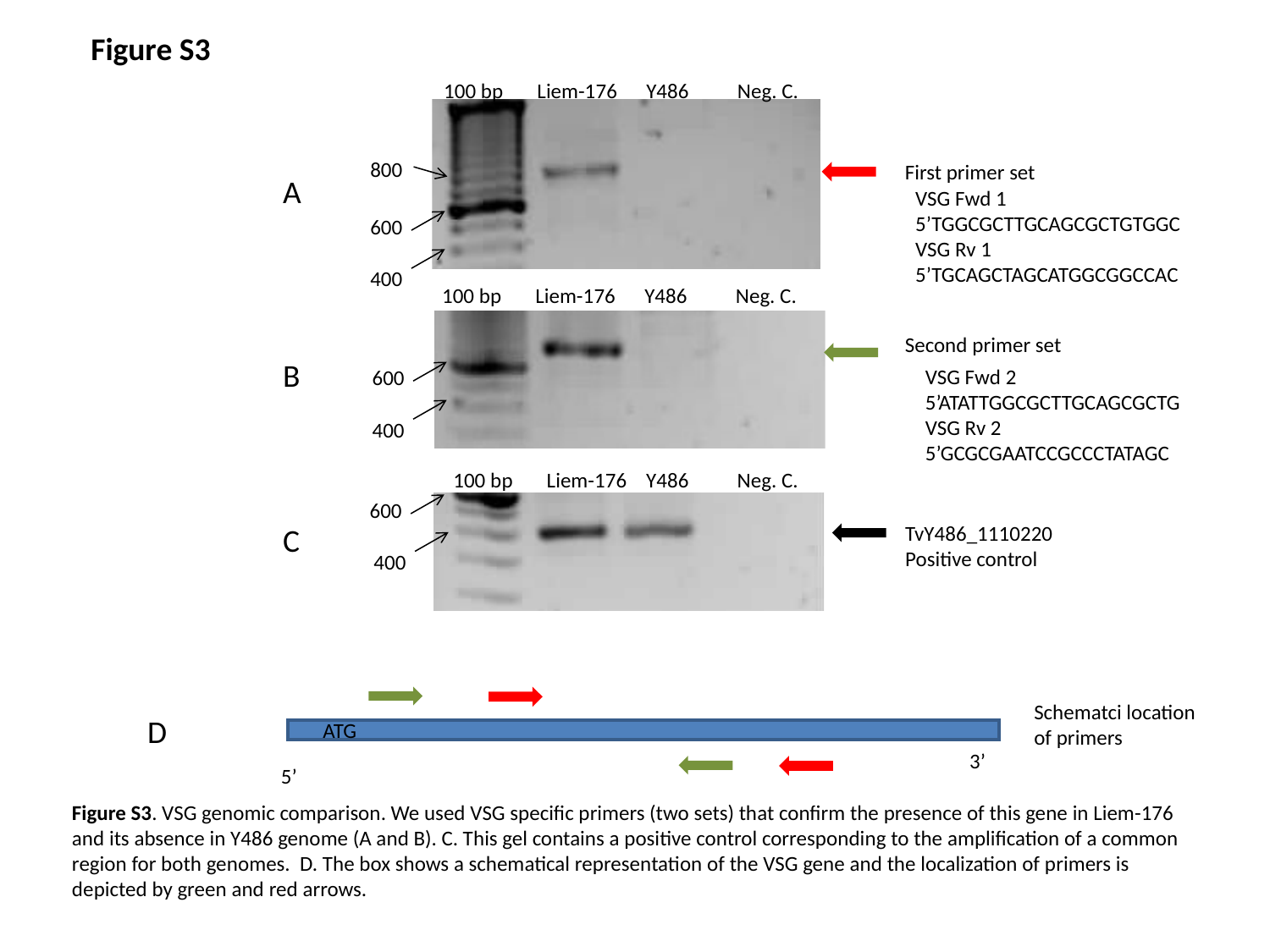

Figure S3
100 bp Liem-176 Y486 Neg. C.
800
600
400
100 bp Liem-176 Y486 Neg. C.
600
400
100 bp Liem-176 Y486 Neg. C.
600
TvY486_1110220 Positive control
400
First primer set
Second primer set
A
VSG Fwd 1
5’TGGCGCTTGCAGCGCTGTGGC
VSG Rv 1
5’TGCAGCTAGCATGGCGGCCAC
B
VSG Fwd 2
5’ATATTGGCGCTTGCAGCGCTG
VSG Rv 2
5’GCGCGAATCCGCCCTATAGC
C
ATG
3’
5’
Schematci location of primers
D
Figure S3. VSG genomic comparison. We used VSG specific primers (two sets) that confirm the presence of this gene in Liem-176 and its absence in Y486 genome (A and B). C. This gel contains a positive control corresponding to the amplification of a common region for both genomes. D. The box shows a schematical representation of the VSG gene and the localization of primers is depicted by green and red arrows.
