# Supplementary material for: Transcriptome analysis of the bloodstream stage from the parasite Trypanosoma vivax
Source: BMC Genomics. 2013 Mar 5;14:149. doi: 10.1186/1471-2164-14-149 (PMC4007602; doi:10.1186/1471-2164-14-149)
Supplement: Additional file 8: Table S5 — rpkm and percentage of total sequence reads corresponding to VSG and tubulin genes in T. vivax and T. brucei. [file 1471-2164-14-149-S8.doc]

**Table S5. Illumina reads mapping.**

| **Illumina reads** | *T. vivax* | *%* | *T. brucei ** | *%* |
| --- | --- | --- | --- | --- |
| VSG | 224379 | 0.70 | 510424 | 5 |
| alpha tubulin | 63766 | 0.18 | 26791 | 0.27 |
| beta tubulin | 112740 | 0.33 | 19837 | 0.20 |

The figures correspond to the number (and percentage) of reads that map in the corresponding CDS (indicated on the left most column).

* Data from RNAseq of *T. brucei* (Siegel et al, 2010).
