# Supplementary figures and images for: Transcriptome analysis of the bloodstream stage from the parasite Trypanosoma vivax
Source: BMC Genomics. 2013 Mar 5;14:149. doi: 10.1186/1471-2164-14-149 (PMC4007602; doi:10.1186/1471-2164-14-149)

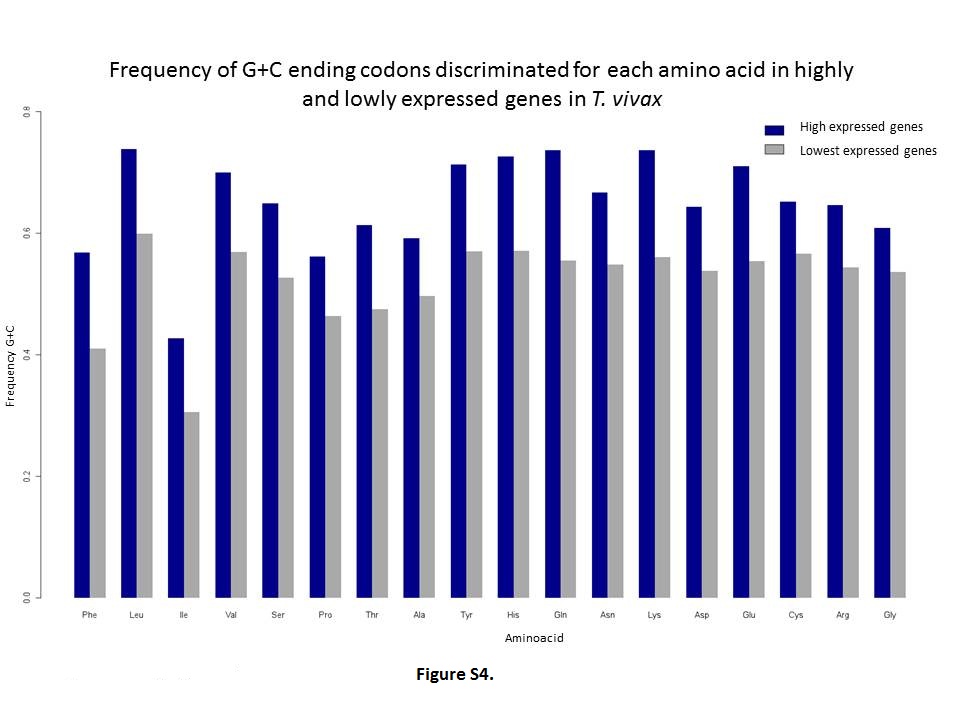

Supplement: Additional file 9: Figure S4 — GC3 content discriminated by amino acid. [file 1471-2164-14-149-S9.jpg]

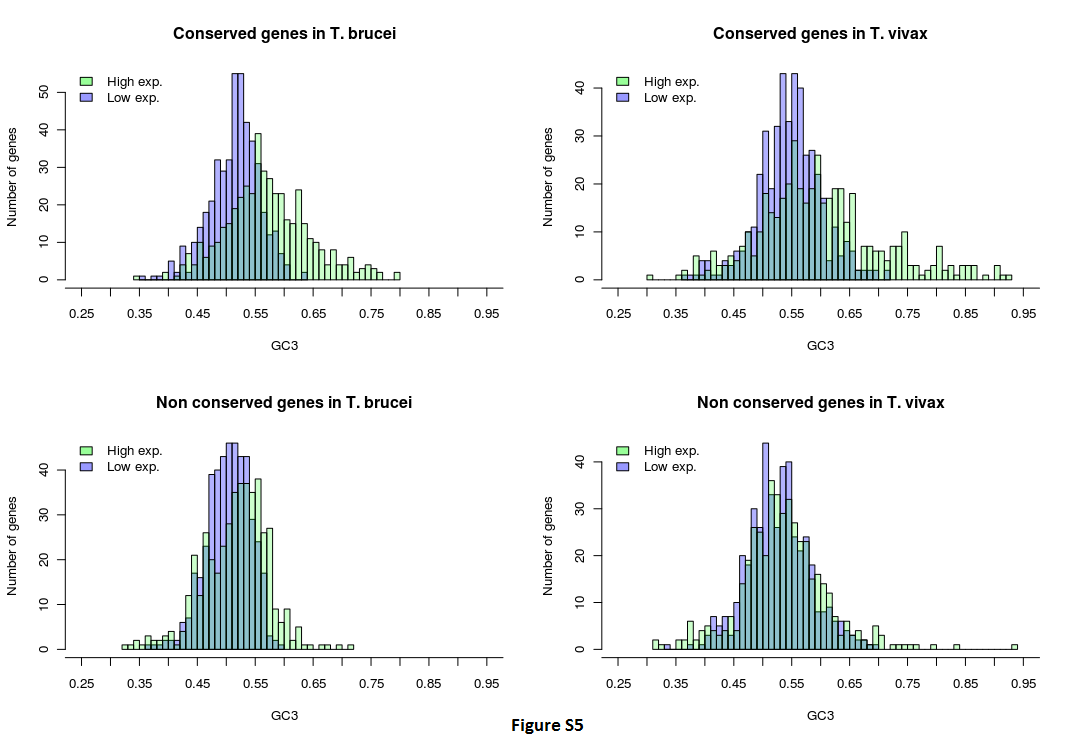

Supplement: Additional file 11: Figure S5 — Comparison of frequencies of G + C ending codons in the most and least expressed genes in T. vivax and T. brucei. The comparison was done between conserved and non conserved orthologous genes (up and low panels). [file 1471-2164-14-149-S11.png]
