# Supplementary material for: Transcriptome analysis of the bloodstream stage from the parasite Trypanosoma vivax
Source: BMC Genomics. 2013 Mar 5;14:149. doi: 10.1186/1471-2164-14-149 (PMC4007602; doi:10.1186/1471-2164-14-149)
Supplement: Additional file 12: Figure S6 — Sequence logo representation of 2nd to 4th trans-splicing sites. [file 1471-2164-14-149-S12.pptx]

## Slide 1
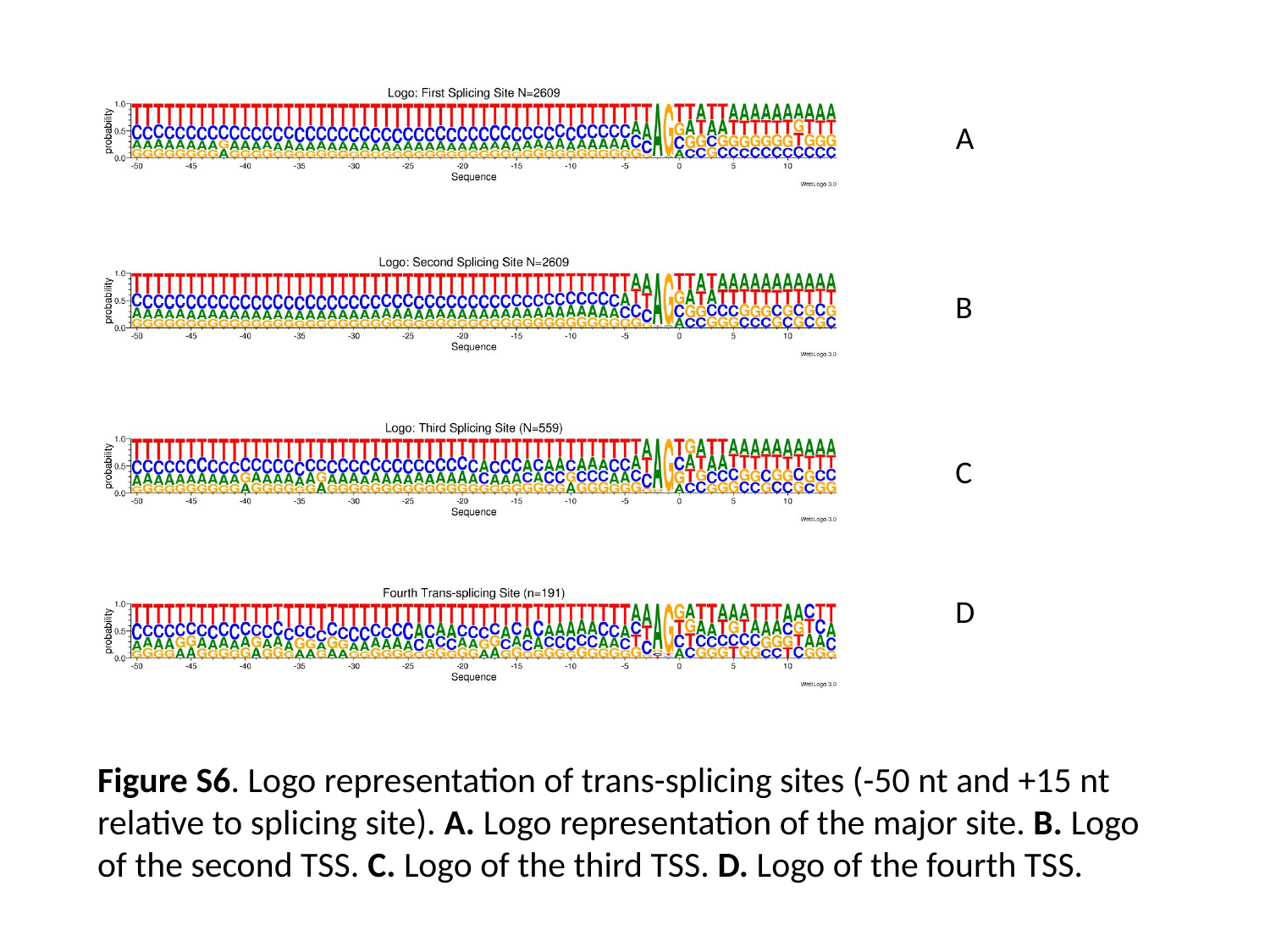

A
B
C
D
Figure S6. Logo representation of trans-splicing sites (-50 nt and +15 nt relative to splicing site). A. Logo representation of the major site. B. Logo of the second TSS. C. Logo of the third TSS. D. Logo of the fourth TSS.
