# Supplementary material for: Transcriptome analysis of the bloodstream stage from the parasite Trypanosoma vivax
Source: BMC Genomics. 2013 Mar 5;14:149. doi: 10.1186/1471-2164-14-149 (PMC4007602; doi:10.1186/1471-2164-14-149)
Supplement: Additional file 13: Figure S7 — Examples of Trans-splicing sites in T. brucei and T. vivax and annotation correction. [file 1471-2164-14-149-S13.docx]

**Figure S7**

**Example 1:**

| **Gene ID** | **Description** | **Reads** | **1st Splicing Site** | **Reads** | **2nd Splicing Site** |
| --- | --- | --- | --- | --- | --- |
| Tb427.08.5810 | mitochondrial carrier protein putative | 67 | 31 | 10 | 34 |
| TvY486_0805310 | mitochondrial carrier protein putative | 12 | -23 |  |  |

**First alignment:**

| Tb427.08.5810 MHALVHFGNEGWLVSTCTWMTEEYVHTVVAGTISGAAGVLLEYPLDTIKVRLQMGGGRYT  TcIL3000.0.57240 -------------------MAEEYALTAVAGTVSGAAGVLLEYPLDTVKVRLQTLGTRYS  TvY486_0805310 -------------------MVPDVCHTLIAGIVSGVAGTVIEYPLDTLKVRLQTCGGRYS  *. : * :** :**.**.::******:***** * **:  Green: Annotated start methionine  Yellow: proposed start methionine |
| --- |
| Tb427.08.5810 AATGTTGCGCAGTGTGCCCACCCGATGCGTCACTTAGCTCCTCTTTTTCTATGCACGCCC  TcIL3000.0.57240 -----------------------------------------------------TTTTTTC  TvY486_0805310 --------------------------------------------------GACCTTCTTT    Tb427.08.5810 TTGTACATTTTGGAAATGAAGGATGGTTAGTTTCCACCTGCACTTGGATGACTGAGGAGT  TcIL3000.0.57240 TTAAAAGCCCGGCAACTG----GCCCCTAGCTCACTGCACCATAGAAATGGCGGAGGAGT  TvY486_0805310 TTACCCACACACCAAC------GTAGACGCCCTGCA-CTGTGTTGCAATGGTGCCCGACG  ** ** * * *** **  Tb427.08.5810 ATGTCCACACGGTTGTAGCGGGTACAATATCTGGCGCGGCTGGTGTATTGCTAGAGTACC  TcIL3000.0.57240 ACGCGCTGACGGCTGTAGCTGGAACGGTATCAGGGGCAGCGGGCGTGTTGCTGGAATATC  TvY486_0805310 TTTGCCATACACTCATTGCGGGCATCGTTTCGGGCGTTGCAGGCACGGTGATTGAGTACC  * ** * ** ** * * ** ** * ** ** ** * ** ** *  Green: annotated start codón  Yellow: Proposed start codon  Red: First trans splicing site  Orange: Second trans splicing site |

**Results after ATG correction:**

| **Gene ID** | **Description** | **Reads** | **1st Splicing Site** | **Reads** | **2nd Splicing Site** |
| --- | --- | --- | --- | --- | --- |
| Tb427.08.5810 | mitochondrial carrier protein putative | 67 | -26 | 10 | -23 |
| TvY486_0805310 | mitochondrial carrier protein putative | 12 | -23 |  |  |

**Alignment after correction:**

| Tb427.08.5810 mitochondrial carrier protein putative  Tb --GAAATGAAGGATGGTTAGTTTCCACCTGCACTTGGATG 38 Tv CACACACCAACG-TAGACGCCCTGCAC-TGTGTTGCAATG 38  * * ** * * * * *** ** * ***  Green: annotated start codón  Red: First trans splicing site  Orange: Second trans splicing site |
| --- |

**Example 2:**

| **Gene ID** | **Description** | **Reads** | **1^st^ Splicing Site** | **Reads** | **2^nd^ Splicing Site** | **Reads/3^rd^ Splicing Site** | **Reads/4^th^ Splicing Site** |
| --- | --- | --- | --- | --- | --- | --- | --- |
| Tb427.08.5800 | Hypothetical protein conserved | 47 | -32 | 32 | -2 | 5/1 | 6/-29 |
| TvY486_0805300 | Hypothetical protein conserved | 5 | 9 | -- | -- | -- | -- |

**First Alignment:**

| Tb427.08.5800 -----MKHKDARGGS-TPYFAITNNKTGEVLLEVAGPLPPTAASPP---PVEEECCGLFN  TcIL3000.0.57230 -----MKHKDGRGQTPPPLFSITNQKTGEVLLEIT-SVPPNNAPLP---PVEEECCGQFN  TvY486_0805300 MPSREMSKNEQNAEQLTPYFAITNQQTGAVLLEITSFGKEFAASTLDEMVVEEECRGLFK  *.::: .. .* *:***::** ****:: *. ***** * *:  Green: Annotated start methionine  Yellow: *T. vivax* proposed start methionine |
| --- |
| Tb427.08.5800 --------------ATGAAACACAAAGATGCTCGCGGTGGTTCGACAC-CGTAC---TTT  TcIL3000.0.57230 --------------ATGAAGCACAAAGATGGACGGGGCCAGACGCCAC-CGCCGCTGTTT  TvY486_0805300 ATGCCTTCTAGAGAAATGAGTAAAAATGAGCAAAATGCGGAACAGCTCACCCCGTACTTT  * * * *** * * * * * * ***  Green: Annotated start codon  Yellow: *T. vivax* proposed start codon |

**Results after ATG correction:**

| **Gene ID** | **Description** | **Reads** | **1^st^ Splicing Site** | **Reads** | **2^nd^ Splicing Site** | **Reads/3^rd^ Splicing Site** | **Reads/4^th^ Splicing Site** |
| --- | --- | --- | --- | --- | --- | --- | --- |
| Tb427.08.5800 | Hypothetical protein conserved | 47 | -32 | 32 | -2 | 5/1 | 6/-29 |
| TvY486_0805300 | Hypothetical protein conserved | 5 | -6 | -- | -- | -- | -- |

**Alignment after correction:**

| Tb427.08.5800 ---------------TCTCCCCTAAACCACCAGTCGCTGTTTTTTTTTGTTTTTGCGGTG  TcIL3000.0.57230 ---------------TAAAATTTTGTCGTCCCACCACGATATCTTTTTCTTTGAGTTGCG  TvY486_0805300 CCCCTCCCACCCCCATTTTCAATTATCTTTTTCTTCTTCTTCTGTTTTTTATGCCTTCTA  * * * * **** * *  Tb427.08.5800 TAGTAATGAAACACAAAGATGCTCGCGGTGGTTCGACAC-CGTAC---TTTGCCATAACG  TcIL3000.0.57230 TAGCAATGAAGCACAAAGATGGACGGGGCCAGACGCCAC-CGCCGCTGTTTTCTATAACC  TvY486_0805300 GAGAAATGA-GTAAAAATGAGCAAAATGCGGAACAGCTCACCCCGTACTTTGCAATAACG  ** ***** * *** * * * * * * *** * *****  Green: annotated start codón  Yellow: *T. vivax* proposed start codon  Red: First trans-splicing site  Orange: Second trans-splicing site  Gray: Third trans-splicing site  Blue: Fourth trans-splicing site |
| --- |

**Example 3**

| **Gene ID** | **Description** | **Reads** | **1^st^ Splicing Site** | **Reads** | **2^nd^ Splicing Site** | **Reads/3^rd^ Splicing Site** | **Reads/4^th^ Splicing Site** |
| --- | --- | --- | --- | --- | --- | --- | --- |
| Tb427.08.6180 | 60S ribosomal protein L26 putative | 3445 | -20 | 414 | -16 | 6/19 | 5/14 |
| TvY486_0805782 | 60S ribosomal protein L26 putative | 296 | -20 | 24 | -11 | -- | -- |

| Tb427.08.6180 MVGIKCRNRRKARRAHFQAPSHVRRILMSAPLSKELRAKYNVRSMPVRKDDEVRVKRGKF  TcIL3000.8.6030 MVGIKCRNRRKARRAHFQAPSHIRRILMSAPLSKELRAKYNVRSMPVRKDDEVRVKRGNF  TvY486_0805780 MPSIKCRNRRKARRAHFQAPSHVRRILMSAPLSKELRAKYNVRSLPVRKDDEVRVKRGAF  * .*******************:*********************:************* *  Green: Annotated methionine start  Yellow: proposed methionine start |
| --- |
| Tb427.08.6180 --CTGGCAATCGGTATTATCATCT-TCCTTTAGCGCCGCACAATCAACCATATATGGTCG  TcIL3000.8.6030 -AATATACACATACACACATATATGTTCAAAAGCTTAACACAATCATCAAC--ATGGTTG  TvY486_0805780 TTTTGGTTGGTTGTTTGCTTGTTTGCTCCAT AGCGCATAAGTGTCCACA--ATGCCAA  * * * * *** ** ** * * ***  Tb427.08.6180 GCATTAAGTGTAGGAACCGCCGAAAGGCCCGTCGCGCACACTTCCAAGCGCCCAGTCATG  TcIL3000.8.6030 GCATCAAGTGCAGGAACCGCCGAAAGGCCCGCCGCGCCCACTTCCAGGCGCCCAGCCACA  TvY486_0805780 GCATAAAGTGTAGGAACCGTCGCAAGGCGCGTCGTGCACACTTTCAGGCCCCGAGTCATG  **** ***** ******** ** ***** ** ** ** ***** ** ** ** ** **  Green: Annotated start Codon  Red: First trans-splicing site  Orange: Second trans-splicing site  Blue: Third and fourth trans-splicing site. |

**Example 4:**

| **Gene ID** | **Description** | **Reads** | **Splicing Site** |
| --- | --- | --- | --- |
| Tb427.03.4810 | hypothetical protein conserved | 41 | 35 |
| TvY486_0304140 | hypothetical protein conserved | 4 | -8 |

**First alignment:**

| Tb427.03.4810 MSFSHFVPPISRRRMFFEDQLDEALSREGSPRLSTSNTVGGADLVSAGAANDETFPFPSH  TcIL3000.0.42620 --------------MFFEEHLDEALSREGSPVIGSGHAANSVN-ISVGGVVDNAFLLASR  TvY486_0304140 -----------**-**--MFFEEHLDEPLSREDSPNLVAG---NGADNVSNGAP----FLFAAR  ****::***.****.** : :. ...: :* *. * :.::  Green: Annotated methionine start  Yellow: *T. brucei* proposed methionine start |
| --- |
| Tb427.03.4810 CAAGAATGTCATTTTCCCATTTTGTACCTCCAATTTCTA----GGAGACGTATGTTTTTTG  TcIL3000.0.42620 TTATTCCTTAATGCTGCCCTTTCATAAGTGTAGCCTCGA----AAAGGTGCATGTTTTTTG  TvY486_0304140 ACAACCGTCAATTACATCCCTTTAAATCCCTTGCTGTGACATTAGGGAGGAATGTTTTTTG  * ** * ** * * * * **********  Green: Annotated start Codon  Red: Trans-splicing site  Yellow: *T. brucei* proposed start codon |

**Results after ATG correction:**

| **Gene ID** | **Description** | **Reads** | **Splicing Site** |
| --- | --- | --- | --- |
| Tb427.03.4810 | hypothetical protein conserved | 41 | -7 |
| TvY486_0304140 | hypothetical protein conserved | 4 | -8 |

**Alignment after correction:**

| Tb --TCCAATTTCTAGGAGACGTATG  Tv GCTGTGACATT AGG-GAGGAATG   * * * **** * ***  Red: Trans-splicing site  Green: Start Codon |
| --- |
